# A role for the vesicle-associated tubulin binding protein ARL6 (BBS3) in flagellum extension in *Trypanosoma brucei*

**DOI:** 10.1016/j.bbamcr.2012.05.007

**Published:** 2012-07

**Authors:** Helen P. Price, Michael R. Hodgkinson, Megan H. Wright, Edward W. Tate, Barbara A. Smith, Mark Carrington, Meg Stark, Deborah F. Smith

**Affiliations:** a Centre for Immunology and Infection, Department of Biology, University of York, Heslington, York YO10 5YW, UK; b Technology Facility, Department of Biology, University of York, Heslington, York YO10 5YW, UK; c Department of Chemistry, Imperial College London, London SW7 2AZ, UK; d Department of Biochemistry, University of Cambridge, Tennis Court Road, Cambridge, UK

**Keywords:** Arf, ADP-ribosylation factor, Arl, ADP-ribosylation factor-like, Arl6ip, Arl6 interacting protein, BBS, Bardet–Biedl syndrome, BBS1, Bardet–Biedl syndrome 1 protein, BSF, bloodstream form, ConA, Concanavalin A, GEF, guanine nucleotide exchange factor, GPCR, G-protein coupled receptor, HRG4, human retinal gene 4, IFT, intraflagellar transport, ITC, isothermal titration calorimetry, MANT, *N*-methylanthraniloyl, MAP2, microtubule associated protein 2, NES, nuclear export signal, NLS, nuclear localisation signal, NMT, myristoyl-CoA:protein *N*-myristoyltransferase, PCF, procyclic form, PCM1, pericentriolar material 1, PFR, paraflagellar rod, PM, plasma membrane, RNAi, RNA interference, RP2, retinitis pigmentosa protein 2, TAP, tandem affinity purification, TiEM, transmission immuno-electron microscopy, *Trypanosoma brucei*, Arl6, BBSome, BBS1, Flagellum, Tubulin

## Abstract

The small GTPase Arl6 is implicated in the ciliopathic human genetic disorder Bardet–Biedl syndrome, acting at primary cilia in recruitment of the octomeric BBSome complex, which is required for specific trafficking events to and from the cilium in eukaryotes. Here we describe functional characterisation of Arl6 in the flagellated model eukaryote *Trypanosoma brucei*, which requires motility for viability. Unlike human Arl6 which has a ciliary localisation, TbARL6 is associated with electron-dense vesicles throughout the cell body following co-translational modification by *N*-myristoylation. Similar to the related protein ARL-3A in *T. brucei*, modulation of expression of ARL6 by RNA interference does not prevent motility but causes a significant reduction in flagellum length. Tubulin is identified as an ARL6 interacting partner, suggesting that ARL6 may act as an anchor between vesicles and cytoplasmic microtubules. We provide evidence that the interaction between ARL6 and the BBSome is conserved in unicellular eukaryotes. Overexpression of BBS1 leads to translocation of endogenous ARL6 to the site of exogenous BBS1 at the flagellar pocket. Furthermore, a combination of BBS1 overexpression and ARL6 RNAi has a synergistic inhibitory effect on cell growth. Our findings indicate that ARL6 in trypanosomes contributes to flagellum biogenesis, most likely through an interaction with the BBSome.

## Graphical abstract

**Figure:**
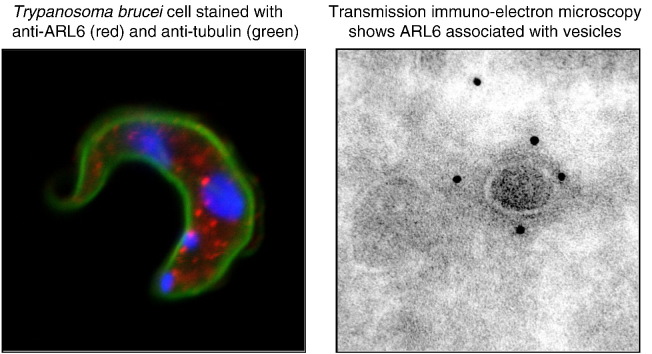

**Highlights:**

► The BBSome-associated protein ARL6 localises to vesicles in *Trypanosoma brucei*. ► *T. brucei* ARL6 is *N*-myristoylated. ► RNAi knockdown causes a decrease in flagellum length but does not affect motility. ► TbARL6 binds to tubulin and has a relatively low affinity for guanine nucleotides. ► The BBSome subunit BBS1 and ARL6 are functionally linked in trypanosomes.

## 1 Introduction

The Arf-like (Arl) proteins are a subfamily of the Arf small GTPases, with diverse roles in vesicle trafficking [1], tubulin folding and microtubule dynamics [2], endosome–lysosome fusion [3] and ciliogenesis [4]. While the functions and effectors of the closely related Arf proteins have been extensively characterised, much less is known about Arl protein interactions and their regulation of fundamental processes within the eukaryotic cell.

Human Arl6 is encoded by *Bbs3*, one of 14 genes in which mutations are implicated in the autosomal recessive clinical disorder Bardet–Biedl syndrome (BBS) [5–7]. The pleiotrophic features of this condition include retinal degeneration, renal abnormalities, polydactyly, obesity, mental retardation and hypogenitalism, many of which are consistent with cilium dysfunction. Comparative genomic analysis has shown that orthologues of Arl6 and the other BBS-associated genes are restricted to ciliated and flagellated organisms, and are absent from species which use flagella only for motility of gametes or zoospores e.g. *Plasmodium falciparum* [7–9]. Expression of Arl6 in *Caenorhabditis elegans* is upregulated in ciliated cells in which the protein is trafficked by intraflagellar transport (IFT) [10]. Recent evidence has shown that GTP-bound human Arl6 can bind BBS1, a subunit of the BBSome complex, and may act in recruitment of this complex to the primary cilium [11]. The BBSome is a stable octomeric complex comprised of 7 Bardet–Biedl syndrome associated proteins (BBS1, 2, 4, 5, 7, 8 and 9) plus a novel protein BBIP10 [12,13]. The exact molecular mechanisms regulated by this complex remain largely unknown but there are marked similarities between the predicted structures of the BBSome components and those of the subunits of the COPII and clathrin cages, indicating a possible function of the BBSome as a coat complex [11]. Further, a recent report indicates that the BBSome subunit BBS7 localises transiently to the nucleus where it binds to the polycomb protein RNF2 and may have a direct role in transcriptional regulation [14].

The BBSome in *C. elegans* appears to mediate the IFT machinery, as loss-of-function mutations in *Bbs7* and *Bbs8* orthologues cause abnormalities in localisation and motility of two IFT proteins, OSM-5/Polaris/IFT88 and CHE-11, resulting in truncated cilia and inhibition of chemotaxis [15]. However, in the majority of systems studied to date, the BBSome is believed to be required for specific trafficking events to and from the cilium rather than for ciliogenesis. Two G-protein coupled receptors (GPCR), somatostatin receptor 3 and melanin-concentrating hormone receptor 1, fail to localise to the cilium of hippocampal neurons in BBSome knockout strains of mice [16]. In contrast, the GPCR dopamine receptor 1, which normally shuttles to and from cilia, can still be trafficked to neuronal cilia in *Bbs2* and *Bbs4* null mice but fails to exit the cilium in response to receptor ligand in these animals [17]. Insertional mutants of *Bbs1*, *Bbs4* or *Bbs7* in the green alga *Chlamydomonas reinhardtii* produce normal length flagella but phototaxis is disrupted due to a defect in retrograde transport from the flagellum leading to accumulation of signalling proteins [18]. No reports have been published to date on the functions of the BBSome or associated proteins in parasitic protozoa or any other pathogenic organisms.

Here we describe functional characterisation of Arl6 in the parasitic protozoan *Trypanosoma brucei*, as part of a comprehensive study of ARF/ARL proteins as substrates of the validated drug target myristoyl-CoA:protein *N*-myristoyltransferase (NMT) [19–22]. We provide experimental evidence that *T. brucei* ARL6 is *N*-myristoylated as predicted by protein sequence analysis. The trypanosome orthologue of Arl6 is found on small electron-dense vesicles throughout the parasite body. The protein is able to associate with tubulin and might therefore act as a link between the BBSome complex and the intracellular microtubule network. Knockdown of TbARL6 is not lethal to the parasite but causes a significant decrease in flagellum length, perhaps indicative of defects in protein trafficking to the flagellum. This observation suggests a degree of evolutionary conservation in the role of this protein and possibly of the BBSome complex in trypanosomes.

## 2 Materials and methods

### 2.1 DNA constructs

All primer sequences are provided in Supplementary Table 1. For protein expression in *Escherichia coli*, a fragment spanning nucleotides 1–570 of the TbARL6 (TriTrypDB ID: Tb927.8.5060) open reading frame was amplified from genomic DNA using primers ARL6-F1 and ARL6-R1 and cloned into plasmid vector pET-YSBLIC3C (pET28a modified for ligation-independent cloning) [23,24] to produce the construct pET-^His^ARL6. For tetracycline-inducible overexpression of C-terminal myc-tagged TbARL6 and N-terminal myc-tagged BBS1 (TriTrypDB ID: Tb09.211.2080) in bloodstream form *T. brucei*, the open reading frames were amplified from genomic DNA using primers ARL6-F2 and ARL6-R2 or BBS1-F1 and BBS1-R1. The products were ligated into plasmid vectors pT7-^MYC-C^ or pT7-^MYC-N^ [22,25]; (obtained from David Horn and Sam Alsford, London School of Hygiene and Tropical Medicine, London, UK) to produce the constructs pTbARL6^MYC^ and pTb^MYC^BBS1. Mutations were introduced into pTbARL6^MYC^ using the GeneTailor Site-Directed Mutagenesis System (Invitrogen) and primers ARL6-F3 and ARL6-R3 (G2A) or ARL6-F4 and ARL6-R4 (T21N) to produce the constructs pTbARL6-G2A^MYC^ and pTbARL6-T21N^MYC^. For RNA interference, a region spanning nucleotides 48–362 of the *T. brucei* ARL6 open reading frame was amplified from genomic DNA using the primers ARL6-F5 and ARL6-R5. The product was digested with *Xba*I and ligated into *Xba*I-digested plasmid vector p2T7Ti (a gift from Doug LaCount, PULSe, Purdue University, West Lafayette, IN, USA) to produce the construct p2T7ARL6. For PTP tagging, the ARL6 open reading frame was amplified from *T. brucei* genomic DNA using primers ARL6-F6 and ARL6-R6, digested with *Apa*I/*Not*I and ligated into digested pC-PTP-NEO (a gift from Arthur Günzl, Department of Genetics and Developmental Biology, University of Connecticut Health Center, Farmington, CT, USA) to produce the construct pTbARL6^PTP^.

### 2.2 Production of recombinant TbARL6 in *E. coli*

The construct pET-^His^ARL6 was introduced into *E. coli* BL21 star and expression of recombinant protein induced with 1 mM IPTG for 4 h at 30 °C. For large-scale protein purification, cells from 5 l of culture were resuspended in 100 ml lysis buffer (300 mM NaCl, 20 mM sodium phosphate pH 7.4, 40 mM imidazole and 1× complete protease inhibitor cocktail, Roche). Cells were lysed by three rounds of sonication then centrifuged at 50,000 *g* for 40 min at 15 °C. Protein purification was performed using Ni^2+^-affinity/size exclusion multi-dimensional liquid chromatography on an ÄKTA Express (GE Healthcare). The clarified lysate was applied at 1 ml/min to a first stage affinity-chromatography column (1 ml HisTrap FF, GE Healthcare) equilibrated in 20 mM sodium phosphate, pH 7.5, 300 mM NaCl and 20 mM Imidazole (Buffer A), before removal of non-specifically bound proteins with 20 column volumes of Buffer A. His-tagged proteins were removed using an isocratic gradient of Buffer A containing 0.5 M imidazole (Buffer B) and the UV_280 nm_ absorbing material was peak-fractionated and collected into a system sample loop. The contents of the sample loop were subsequently applied at 1 ml/min to a second stage size exclusion-chromatography column (Superdex200 16/60, GE Healthcare) equilibrated in 20 mM sodium phosphate, pH 7.5, 150 mM NaCl (Buffer C) and then eluted using a 1.2 column volume isocratic gradient of Buffer C. Fractions (1.8 ml) of UV_280 nm_ absorbing material were collected and analysed for specific protein content by SDS-PAGE.

### 2.3 Antibody production

Polyclonal antibodies were produced from two rabbits (Eurogentech, 87 day Classic protocol). Antibodies were purified using a 1 ml NHS-activated HP column (GE) coupled to 5 mg of recombinant TbArl6 protein. Following column equilibration with 10 ml binding buffer (20 mM sodium phosphate pH 7 and 150 mM NaCl), 15 ml rabbit serum was loaded onto the column at 0.3 ml/min. Unbound sample was removed with a 5 ml wash with binding buffer. Elution was then performed using elution buffer at low pH (0.1 M glycine pH 2.7 and 0.5 M NaCl). Fractions of 0.5 ml were collected directly into tubes containing 50 μl 1 M Tris–HCl pH 9.0 and analysed by SDS‐PAGE. Peak fractions were pooled and tested by immunoblotting.

### 2.4 Parasite culture

The *T. brucei brucei* BSF strain Lister 427 was maintained *in vitro* at 37 °C with 5% CO_2_ in HMI-9 medium containing 2 μg/ml Geneticin (Invitrogen) as described [26]. The *T. brucei brucei* procyclic strain 449 was maintained *in vitro* at 26 °C in SDM-79 medium containing 25 μg/ml phleomycin [26]. All culture media contained 10% tetracycline-free fetal bovine serum (Autogen Bioclear).

### 2.5 Protein analysis

Immunoblotting of total parasite lysates was performed as described previously [22,27]. Primary antibodies used were: rabbit anti-TbARL6 (1:500 dilution); rabbit anti-TbNMT [19] (1:500 dilution); mouse anti-myc (1:2000 dilution, Invitrogen); and mouse monoclonal L13D6 against PFR1/2 (1:500 dilution, a gift from Keith Gull, Sir William Dunn School of Pathology, University of Oxford, UK). For subcellular fractionation, parasites were centrifuged at 800 *g* for 10 min at 20 °C, then washed in PBS. For cytosolic (S100) and particulate membrane-containing (P100) fractions, cells were subjected to hypotonic lysis on ice for 1 h in 10 mM Tris pH 7.5 containing 1× Complete protease inhibitor cocktail (Roche), 7.5 μM Pepstatin A and 5 μM E-64d. Samples were centrifuged at 100,000 *g* for 30 min at 4 °C, then supernatant (S100) and pellet (P100) fractions stored in Laemmli buffer. Previous studies have shown that *T. brucei* cytoskeletal and flagellar fractions can be obtained by subjecting cells to detergent/NaCl extraction [27,28]. Parasites were resuspended in PEME (100 mM PIPES, 2 mM EGTA, 0.1 mM EDTA and 1 mM MgSO_4_, pH 6.9) containing 1% Nonidet P40, 1× Complete protease inhibitor cocktail (Roche), 7.5 μM Pepstatin A and 5 μM E-64d, incubated on ice for 10 min, then centrifuged at 15,000 *g* for 15 min at 4 °C. Pellets were either washed twice in PEME and resuspended in Laemmli buffer (NP40-insoluble fraction containing cytoskeleton) or further extracted in PEME containing 1 M NaCl, 200 μg/ml DNaseI, 50 μg/ml RNaseA and protease inhibitors as above. Samples were incubated on ice for 10 min, centrifuged as above, salt extraction repeated once, then pellets washed twice in PEME. Pellets were resuspended in Laemmli buffer (NP40/1 M NaCl-insoluble fraction containing flagella). Total cell lysates and subcellular fractions from the equivalent of 1 × 10^7^ cells per sample were analysed by immunoblotting and probed with antibodies as above.

### 2.6 Myristate tagging and fluorescent labelling of TbARL6

Parasites were metabolically labelled by the addition of 100 μM myristic acid or the myristate analogue YnC12 [29] to *T. brucei* BSF (1 × 10^6^/ml in HMI-9) or PCF (5 × 10^6^/ml in SDM-79). Cells were then grown for 8 h at 37 °C with 5% CO_2_ (BSF) or 18 h at 26 °C (PCF) before harvesting. Parasites were lysed in ice-cold RIPA buffer (50 mM Tris pH 7.4, 1% (v/v) NP-40, 1% (w/v) sodium deoxycholate, 150 mM NaCl, 0.5% (w/v) SDS and 1× Complete protease inhibitor cocktail (Roche)), sonicated, then centrifuged at 15,000 *g* for 30 min at 4 °C. Lysate protein concentrations were determined by the DC protein assay (BioRad). Protein lysates (0.5 mg total protein) from myristate-labelled and YnC12-labelled cells were incubated with 10 μl of anti-TbARL6 with rotation overnight at 4 °C. Approximately 30 μl packed Fast Flow Protein A Sepharose beads (GE Healthcare) were added and the lysates rotated for 2 h at 4 °C. Beads were washed twice with ice-cold RIPA buffer, twice with 0.1% (w/v) SDS/PBS then resuspended in 50 μl PBS for on-bead click labelling. Click reagents (TAMRA-capture reagent Az-TB, CuSO_4_, TCEP and TBTA) were prepared and premixed as described previously [29], added to the beads and left on ice with occasional gentle vortexing for 90 min. SDS was added to a final concentration of 2% and the beads heated for 10 min at 95 °C. The solution was removed and the proteins precipitated (CHCl_3_/MeOH), then resuspended in 1× sample loading buffer with beta-mercaptoethanol (NuPAGE LDS sample buffer) for gel and blot analysis.

Samples were separated by SDS-PAGE and the gel washed briefly in water for in-gel fluorescent imaging. Gels were scanned with Cy3 filters to detect the TAMRA fluorophore using an Ettan DIGE scanner (GE Healthcare). Proteins were transferred to PVDF membrane, blocked (5% milk in TBS 0.1% Tween-20, 40 min at room temperature), washed, and reimaged for fluorescent signal. The membrane was then incubated with anti-TbARL6 (1:300 in blocking solution) for 90 min, then probed with goat anti-rabbit-HRP secondary (Invitrogen) (1:5000 in blocking solution) for 30 min. Proteins were visualised with Luminata Crescendo Western HRP substrate (Millipore).

### 2.7 Microscopy

Indirect immunofluorescence assays were performed as described [22] using rabbit anti-TbARL6 (1:100, described in Section 2.3), mouse monoclonal antibody TAT1 against *T. brucei* α-tubulin (1:200, a gift from Keith Gull, as in Section 2.5), mouse monoclonal L13D6 (as in Section 2.5, 1:50), rabbit anti-TbRab5A (1:250, a gift from Mark Field, Department of Pathology, University of Cambridge, UK) and mouse anti-myc (1:250, Invitrogen) with detection using Alexa Fluor 488 and 594 conjugated secondary antibodies (Invitrogen). Staining with FM4-64FX (Invitrogen) and Alexa Fluor 488-conjugated ConA (Invitrogen) were performed as described previously [21,22]. Samples were visualised by confocal microscopy as described previously [21].

Transmission electron microscopy was performed as described [30]. For transmission immuno-electron microscopy, BSF cells in culture medium were diluted in a 1:1 ratio with 8% formaldehyde (w/v)/0.4% glutaraldehyde (w/v) in 100 mM sodium phosphate and incubated for 15 min. Following centrifugation, cells were sequentially treated with 4% formaldehyde (w/v)/0.2% glutaraldehyde (w/v) in 100 mM sodium phosphate buffer for 15 min, 0.1% glycine (w/v) in 100 mM PBS for 20 min, then in 1% gelatin in pure water for 30 min. Cells were then treated with 10% gelatin in pure water and the cell pellet spun down, cut into pieces and infused with 2.3 M sucrose in PBS overnight at 4 °C. Samples were mounted on Leica cryo-holders and frozen in liquid nitrogen before sectioning (approximately 90 nm thickness) on a Leica Ultracut with cryochamber. Sections were transferred to copper grids, then inverted onto drops of liquid on parafilm for immunogold labelling. The grids were incubated in 50 mM glycine/PBS for 5 min, in blocking solution (10% goat serum/PBS) for 20 min, then in primary antibody (rabbit anti-TbARL6 as in Section 2.3 or mouse anti-myc, Invitrogen) diluted 1:25 in blocking solution for 1 h. Samples were washed five times in blocking buffer, then incubated in 10 nM gold conjugated goat anti-rabbit or anti-mouse IgG (Agar Scientific) diluted 1:10–1:40 in blocking buffer for 45 min, then washed five times in blocking buffer. Cells were then fixed in 1% glutaraldehyde in 100 mM sodium phosphate buffer for 10 min and washed five times in pure water. Samples were incubated with 2% methyl cellulose/0.4% uranyl acetate on parafilm placed on an ice-chilled metal block in the dark for 10 min. Viewing was at 120 kV with a Tecnai 12 BioTwin (FEI) and images were captured with a SIS Megaview III digital camera.

### 2.8 Expression of myc-tagged ARL6 and BBS1

The constructs pTbARL6^MYC^, pTbARL6-G2A^MYC^, pTbARL6-T21N^MYC^ and pTb^MYC^BBS1 (*Not*I digested) were transfected into mid-log phase *T. brucei* BSF Lister 427 using the Nucleofector® system as described [31] and transformants selected with 10 μg/ml hygromycin. Expression of tagged protein was induced in stable cell lines by incubating parasites in 1 μg/ml tetracycline for 24 h. Parasite growth was analysed using a Beckman Coulter counter.

### 2.9 RNA interference of ARL6

The construct p2T7ARL6 (*Not*I digested) was transfected into mid-log *T. brucei* BSF Lister 427 by nucleofection as in Section 2.7 and transformants selected with 2.5 μg/ml phleomycin. Production of TbARL6-specific double-stranded RNA was induced by incubating stable cell lines in 1 μg/ml tetracycline for 24 h. Knockdown of ARL6 expression was monitored by immunoblotting with anti-TbARL6 antibody. Stable ARL6 RNAi cell lines were also transfected with *Not*I digested pTb^MYC^BBS1, transformants selected by the addition of 10 μg/ml hygromycin and cell lines analysed as in Section 2.7. Cell body and paraflagellar rod (PFR) lengths were analysed by performing indirect immunofluorescence on cells treated with 1% Triton X-100 at room temperature for 5 min before staining with mouse monoclonal L13D6 or TAT1 (as in Section 2.6) and detection using Alexa Fluor 488-conjugated goat anti-mouse IgG and IgM (Invitrogen). Cell body (posterior end of the body to the flagellum tip) and PFR lengths (100 per sample) were measured from acquired images using LSM 510 version 3.2 software (Zeiss). Statistical analysis (1 way ANOVA) was performed using GraphPad Prism 4.

### 2.10 Mouse infections

Swiss outbred (CD-1) mice were infected by i.p. injection with 2 × 10^5^ parasites of the parental or TbARL6 RNAi lines as in Section 2.8, grown in the absence or presence of tetracycline for 4 h prior to infection. Mice given tetracycline-treated cells were given doxycycline in drinking water. Parasitaemia was measured at 48 h and 72 h by microscopy analysis of tail-cut blood samples and the experiment terminated at the later timepoint.

### 2.11 Tandem affinity purification

Log phase *T. brucei* procyclic strain 449 cells were transfected with *Bsm*I-digested pTbARL6^PTP^ by nucleofection as in Section 2.7, replacing one endogenous allele of ARL6 with a C-terminal PTP tagged copy of the ARL6 open reading frame. Transformants were selected with 25 μg/ml hygromycin. Once stable cell lines had been established, tandem affinity chromatography was performed as described previously [32]. Briefly, a crude extract was prepared from 9 l of culture (approximating 1 × 10^11^ cells) by resuspension of cells in 15 ml of lysis buffer (150 mM sucrose, 150 mM KCl, 20 mM potassium glutamate, 20 mM Hepes–KOH pH 7.7, 3 mM MgCl_2_, 10 mg/ml leupeptin, and 0.5% DTT) and passaging through a French press at 4 °C. The lysate was subjected to IgG affinity chromatography and bound complexes were eluted by incubation with AcTEV protease. The eluate was then subjected to anti-ProtC affinity chromatography. Proteins were eluted by the addition of EGTA/EDTA to inhibit calcium-dependent binding. The final eluate was concentrated by binding to 10 μl of hydrophobic StrataClean resin (Stratagene) then released into Laemmli SDS-PAGE loading buffer. Samples were separated by SDS-PAGE and stained with Coomassie. Protein bands were excised and identified by MS/MS analysis of trypsin digests on a 4700 Proteomics Analyser (Applied Biosystems). The mass spectral data were submitted to database searching against the NCBInr database using a locally running copy of the Mascot software (Matrix Science) through a GPS Explorer (Applied Biosystems) interface. The data shown are representative of 4 independent biological replicates.

### 2.12 Tubulin overlay assay

Overlay assays were performed as described [33] using TbARL6 recombinant protein (described in Section 2.2) and negative controls: recombinant human Ras (R9894, Sigma) and bovine serum albumin (New England Biolabs). Proteins (1 μg per lane) were subjected to SDS-PAGE and transferred to nitrocellulose. A duplicate gel was stained with Coomassie Blue. Blots were incubated in TBS/1% Tween-20 for 45 min at room temperature, then in 8 μg/ml porcine tubulin (Sigma) in TBS/0.2% Tween-20 overnight at 4 °C. Blots were washed in TBS/0.2% Tween-20, blocked in 5% milk/TBS/0.2% Tween-20 and probed with mouse monoclonal antibody TAT1 (α-tubulin, 1:10,000 dilution), mouse monoclonal clone 6-11B-1 (acetylated α-tubulin, 1:10,000 dilution, Sigma) or mouse monoclonal TUB 2.1 (β-tubulin, 1:1000, Sigma).

### 2.13 Microtubule binding assay

In order to determine if TbARL6 was able to bind to microtubules *in vitro*, a Microtubule Binding Protein Spin-Down Assay kit (Cytoskeleton, Inc.) was used, following the manufacturer's instructions. All buffers containing polymerised tubulin were supplemented with 20 μM Taxol. Briefly, 2.5 μg of pure TbARL6 protein was incubated for 30 min at room temperature with a suspension of freshly polymerised microtubules (equivalent of 2 μM of tubulin dimer) in the presence of 10 mM MgCl_2_ and 4 mM GDP or GTP. Control samples were also prepared using 2.5 μg of MAP2 or BSA instead of TbARL6, or with the omission of either the test protein or microtubules from the reaction mix. Following incubation, samples were placed onto a layer of Cushion Buffer (80 mM PIPES, pH 7.0, 2 mM MgCl_2_, 1 mM EGTA, and 60% glycerol) and centrifuged at 100,000 *g* for 40 min. The uppermost layer of supernatant (soluble fraction) and pellet (insoluble fraction) were subjected to SDS-PAGE and stained with Coomassie Blue. In addition, the effects of TbARL6 on tubulin polymerisation *in vitro* were analysed by measuring turbidity as described previously [34].

### 2.14 Fluorescence spectroscopy

All samples were diluted in 50 mM KHPO_4_ (pH 7.4), with 10 mM MgCl_2_ and 12.5 μm unlabelled GDP/GTP (Sigma) or 5 μM *N*-methylanthranil acid (mant) labelled GDP/GTP (Invitrogen). Measurements were recorded on a Fluoromax-4 spectrophotometer (Horiba Jobin-Yvon Ltd, Stanmore, UK). Tryptophan fluorescence spectra (5 μM protein) were measured at an excitation of 278 nm and emission range of 300–450 nm. Mant fluorescence spectra were measured at an excitation of 350 nm and emission range of 350–550 nm. Kinetic data were collected at an emission of 447 nm (GDP) or 445 nm (GTP) using 5 μM of mant labelled nucleotide and a range of TbARL6 concentrations between 0.2 and 14 μM. Nucleotide exchange was studied by loading 5 μM of protein with 5 μM mant labelled nucleotide for 5 min, then adding a 100-fold excess of unlabelled nucleotide and collecting fluorescence data every 10 s for a further 20 min. K_D_ and κ_off_ values were calculated by non-linear regression using GraphPad Prism 4. Values shown represent the mean from three independent experiments.

## 3 Results

### 3.1 *T. brucei* ARL6 orthologue

We first identified the *T. brucei* ARL6 orthologue in a bioinformatic search for downstream targets of the validated drug target myristoyl-CoA:protein *N*-myristoyltransferase (NMT) [19,22,35]. The constitutively expressed *TbARL6* gene encodes a protein of 21 kDa, which is predicted to be *N*-myristoylated by virtue of the well-conserved N-terminal consensus for *N*-myristoylation [36]. This is in contrast to ARL6 protein sequences from other species including humans [37] which have an N-terminal glycine residue but are not predicted by available algorithms to be *N*-myristoylated. Similar to human BBS7, the TbARL6 protein does not contain a nuclear localisation signal (NLS) but does have a leucine-rich nuclear export signal (NES) motif (residues 139–147), using the prediction software programs NLStradamus and NetNES 1.1 [38,39]. Using the same programs, human Arl6 also has a predicted NES (residues 9–13) but no NLS.

### 3.2 TbARL6 associates with vesicles throughout the parasite body

In order to investigate subcellular localisation in *T. brucei*, we raised a polyclonal antibody against full length recombinant protein. TbARL6 fused to a C-terminal His tag was readily expressed as a soluble protein in *E. coli* and purified by affinity purification (Supplementary Fig. 1A). Serum from a rabbit inoculated with recombinant TbARL6 detected a band of approximately 22 kDa on immunoblots of total *T. brucei* lysates (Supplementary Fig. 1B). Further, only the 22 kDa band was detected in cell lysates by affinity purified antibody (Supplementary Fig. 1B). Although we cannot rule out the possibility that other ARF family proteins could be detected by the antibody, the most closely related protein to ARL6 encoded by the *T. brucei* genome is the Golgi protein ARL1, which shares only 37% identity [22], while TbARF1 shares less than 30% identity with TbARL6.

**Fig. 1:**
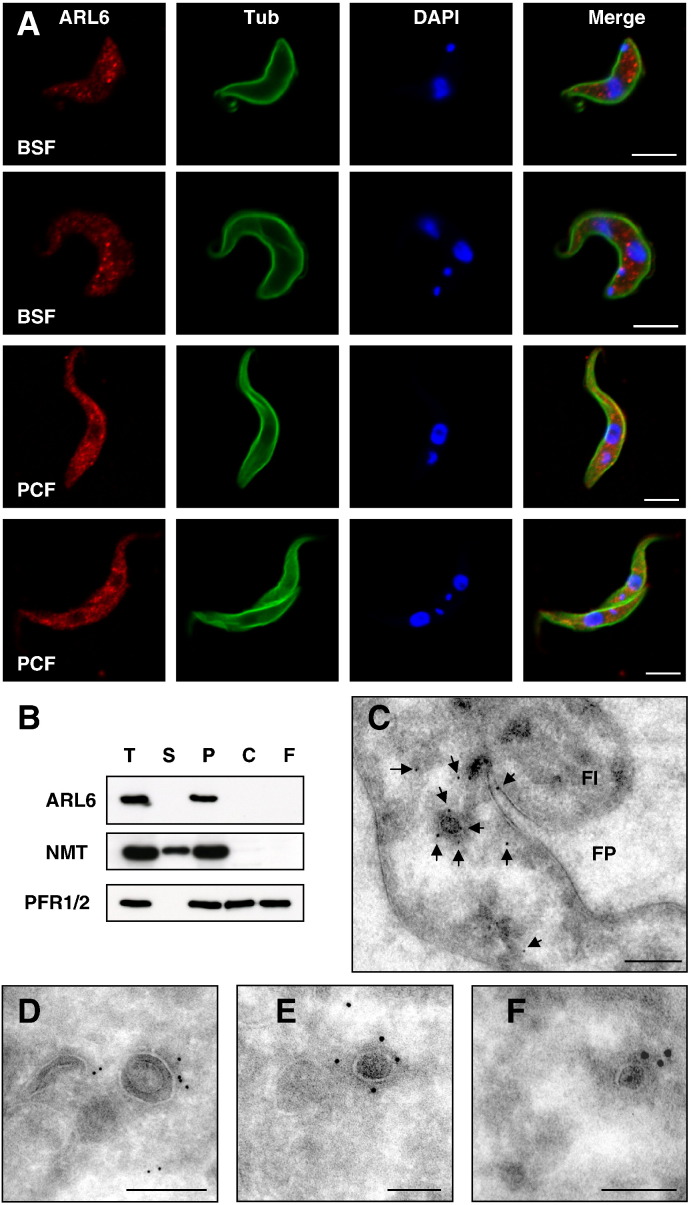
Subcellular localisation of *T. brucei* ARL6. (A) Immunofluorescence analysis of *T. brucei* bloodstream form (BSF) line Lister 427 and insect procyclic form (PCF) line 449. Representative images of non-dividing (one nucleus and one kinetoplast) and dividing (two nuclei and two kinetoplasts) cells are shown for each life cycle stage. Cells were probed with anti-TbARL6 (red) and anti-α tubulin (green) and co-stained with DAPI (blue). Bar, 5 μm. (B) Subcellular fractionation of TbARL6. Prepared subcellular fractions from *T. brucei* BSF line Lister 427 (equivalent of 1 × 10^7^ cells per lane) were separated by SDS-PAGE, transferred to PVDF membrane and the resulting immunoblot probed with anti-TbARL6, anti-NMT and anti-PFR1/2. T, total cell lysate, S, cytosolic S100 fraction, P, particulate membrane-containing P100 fraction, C, NP40-insoluble fraction (containing cytoskeleton), F, NP40/1M NaCl fraction (containing flagella). (C–F) Transmission immuno-electron micrographs of BSF line Lister 427 probed with anti-TbARL6 and detected with 10 nm colloidal gold conjugated goat-anti-rabbit. Fl, flagellum; FP, flagellar pocket. Gold particles are indicated in (C) by black arrows. Bar, 200 nm (C, D) or 100 nm (E, F).

Immunofluorescence assays using the antibody showed that ARL6 is present in a punctate distribution throughout the cell body in both bloodstream form (BSF) and insect procyclic form (PCF) *T. brucei* and has the same localisation in non-dividing and dividing cells (Fig. 1A). Some staining was observed at the periphery of the nucleus, consistent with the presence of a NES motif in the protein. There was no obvious co-localisation between TbARL6 and the early endosomes, as shown in cells incubated with fluorescent ConA at 15 °C (Supplementary Fig. 2A). Localisation was investigated further by subcellular fractionation, separating cells into cytosolic (S100) and particulate membrane-containing (P100) fractions. The *T. brucei* cytoskeleton and flagella were also isolated by treatment with detergent and salt. The NP40-insoluble fraction contains all components of the tubulin-based cytoskeleton but further extraction with salt solubilises the subpellicular microtubules, therefore the NP40/1M NaCl-insoluble fraction contains only the highly insoluble flagella. TbARL6 protein was found in the membrane-containing P100 fraction of BSF cell lysate but not in the cytosolic S100 fraction, NP40-insoluble or NP40/1M NaCl-insoluble fractions (Fig. 1B). This suggests that the protein associates with cellular membranes but is not an integral part of the cytoskeleton, consistent with its reported absence from the flagellar proteome [28]. Intracellular localisation was studied at high resolution by transmission immuno-electron microscopy (TiEM) (Fig. 1C–F). Endogenous TbARL6 was detected throughout the cell, particularly in association with electron-dense vesicles of less than 100 nm diameter (Fig. 1C, E, and F) and to a lesser extent with larger structures of approximately 200 nm diameter (Fig. 1D). Negligible levels of gold labelling were detected in negative-control samples without primary antibody (Supplementary Fig. 3A).

When TbARL6 was overexpressed with a C-terminal myc tag, a punctate pattern was also observed by immunofluorescence, although the foci were larger than for endogenous TbARL6 (Supplementary Fig. 2B, top panel). Unlike endogenous TbARL6, the myc-tagged protein showed some co-localisation with the early endosomes as visualised using an antibody against *T. brucei* Rab5 (Supplementary Fig. 2B). This may represent either mislocalisation due to the epitope tag or slower trafficking of the over-expressed protein. TiEM images show that the tagged protein is associated with circular structures (Supplementary Fig. 3) similar in morphology to the 200 nm diameter structures found adjacent to a minor population of the endogenous protein (Fig. 1D and data not shown). In contrast, a G2A mutant form of the protein lacking the N-terminal glycine residue (the myristate acceptor) mislocalised to the plasma membrane (Supplementary Fig. 2B). This mutant isoform was readily expressed even in the absence of tetracycline, showing some leakiness of the inducible promoter system. Unlike the wild-type, the G2A mutant protein appeared to be a target for proteolysis at its new site in the cell, as shown by immunoblotting (Supplementary Fig. 2C). Another mutant form of TbARL6, T21N (a critical residue for nucleotide binding found at the P-loop) localised partially at the plasma membrane and partially in a punctate distribution, a subpopulation of which co-localises with Rab5 (Supplementary Fig. 2B).

### 3.3 Evidence that TbARL6 is N-myristoylated

**Fig. 2:**
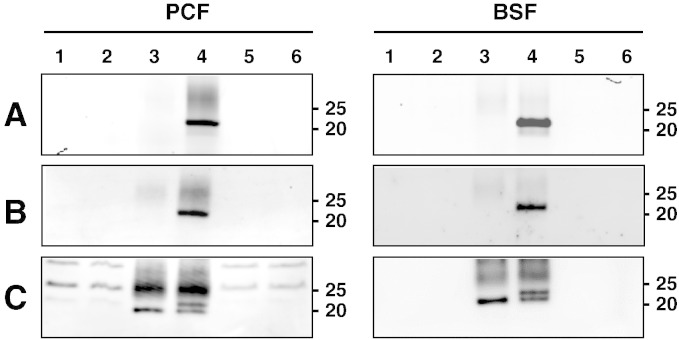
Myristate tagging and fluorescent labelling to show *N*-myristoylation of TbARL6. (A–C) *T. brucei* insect procyclic form (PCF) line 449 and bloodstream form (BSF) line Lister 427 were metabolically labelled with myristic acid or the myristate analogue YnC12. Immunoprecipitation was performed on cell lysates by the addition of rabbit anti-TbARL6 and Protein A Sepharose beads, and captured proteins were subjected to on-bead click labelling with the fluorophore TAMRA. Samples were eluted, separated by SDS-PAGE, transferred to PVDF membrane and fluorescence was detected using an Ettan DIGE scanner (GE Healthcare). In-gel (A) and in-blot (B) fluorescence are shown for PCF (left panels) and BSF (right panels) lysates. (C) Immunoblots were probed with anti-TbARL6. 1, 2, Total lysate from myristic acid-labelled and YnC12-labelled cells respectively (20% of fraction loaded), 3, 4, Elution from beads after immunoprecipitation of lysate from myristic acid-labelled and YnC12-labelled cells respectively (100%), 5, 6, Supernatant following immunoprecipitation for myristic-acid labelled and YnC12-labelled cells respectively (20%).

We tested the ability of TbARL6 to be *N*-myristoylated by metabolic labelling of parasites with the alkynyl-myristate YnC12 [40], which acts as a myristic acid analogue in cells and can be captured by copper-catalysed click chemistry in combination with fluorescent probes [29]. TbARL6 was captured from parasite lysates by immunoprecipitation and labelling of YnC12-tagged proteins was carried out on-bead via a click chemistry reaction between the alkyne functionality and fluorescent reagent Az-TB. Eluted proteins were analysed by SDS-PAGE and immunoblotting as shown in Fig. 2. A strong fluorescent signal was observed at the predicted molecular weight of TbARL6 (approximately 21 kDa) following immunoprecipitation from *T. brucei* PCF and BSF lysates labelled with YnC12 (Fig. 2A and B, in-gel and in-blot fluorescence), indicating that TbARL6 is labelled by this alkynyl myristate analogue under the experimental conditions used. No signal was detected following immunoprecipitation in the myristic acid control, in the total lysates prior to fluorescent labelling or in the supernatant fraction following capture of TbARL6 to the beads. Immunoblotting for TbARL6 confirmed the presence of immunoprecipitated protein in both YnC12 and myristic acid samples (Fig. 2C). A number of background bands were observed, particularly in the PCF samples. However, two specific bands were observed for TbARL6 in YnC12-labelled PCF and BSF lysates following immunoprecipitation: the 21 kDa band and an additional higher molecular weight band which likely corresponds to the protein carrying the Az-TB label (mw of ~ 1000), as shown in previous analysis of cholesterol-modified Sonic hedgehog protein [41]. Comparison of the relative intensities of the two bands gives an approximate tagging-labelling efficiency for TbARL6 of 50% using this methodology.

### 3.4 Depletion of TbARL6 by RNA interference

**Fig. 3:**
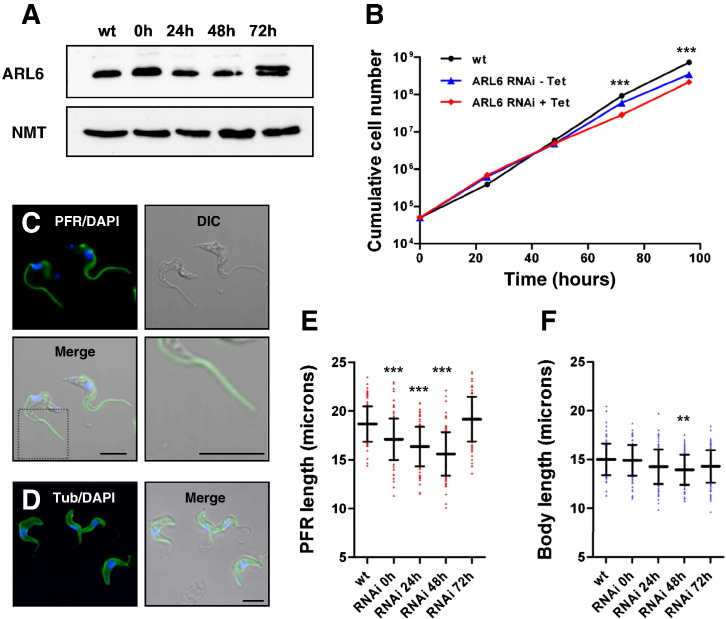
TbARL6 knockdown by RNA interference (A) Total cell lysates (5 × 10^6^ cells/lane) from BSF parental line Lister 427 (wt) and cell line 427/p2T7ARL6 (RNAi) grown in the presence of tetracycline for 0–72 h were immunoblotted and probed with anti-TbARL6 and anti-NMT to monitor equal sample loading. (B) Cumulative growth of BSF parental line Lister 427 (wt) and transfected line 427/p2T7ARL6 (RNAi) in the absence and presence of tetracycline, monitored over a 5 day time course. Mean values (± SD) are plotted (n = 3), error bars not visible. (C, D) Immunofluorescence analysis of cell line 427/p2T7ARL6 grown in the presence of tetracycline for 24 h, probed with anti-PFR1/2 (C) or anti-α-tubulin (D) (shown in green) and co-stained with DAPI (blue). Bar, 5 μm. The bottom right panel in (C) is an enlarged view (× 2.5) of the region marked by a grey box in the bottom left panel. (E, F) Paraflagellar rod length (E) and cell body length (F) in BSF parental line Lister 427 (wt) and cell line 427/p2T7ARL6 (RNAi) grown in the presence of tetracycline for 0–72 h. Mean values are shown as horizontal lines (± SD) (n = 100). Statistical analyses were performed using 1 way ANOVA. Asterisks represent statistical significance compared to the wild-type (wt) sample (** = p < 0.01, *** = p < 0.001). All data shown are representative of experiments using at least 3 independent RNAi clones.

The functions of TbARL6 were investigated by disrupting expression in *T. brucei* BSF parasites using tetracycline-inducible RNA interference (RNAi). TbARL6 protein levels were analysed in 6 independent RNAi parasite clones by immunoblotting and densitometry using TbNMT as a loading control (one representative example shown in Fig. 3A). The protein level was reduced in induced cells but in all cases remained at detectable levels throughout a 96 hour time course (Fig. 3A and data not shown) demonstrating that RNAi knockdown was incomplete. TbARL6 in the RNAi cell line shown in Fig. 3A decreased to 55% and 26% at 24 and 48 h post-induction respectively, compared to the level in uninduced cells. TbARL6 protein persistently increased at either 72 or 96 h, indicative of the emergence of escape mutants resistant to tetracycline induction, a common feature of *T. brucei* RNAi studies [35,42]. An additional protein band (approximately 24 kDa) of unknown identity was also detected by the ARL6 antibody in some but not all clones at later timepoints (Fig. 3A).

There was no evidence in any of the analysed parasite clones of widespread cell death and no decrease in growth until late timepoints (72 and 96 h) when there was a modest but statistically significant reduction in growth in induced cells compared to uninduced cells (p = < 0.001) (Fig. 3B). We cannot conclude that the observed defects in growth late in the time course were due to a decrease in ARL6 protein because of the apparent emergence of rescue mutants but we are able to surmise from our data that a moderate decrease in TbARL6 has no effect on parasite growth within 48 h of induction.

Changes in cell morphology, in particular in assembly of the parasite flagellum, were investigated by immunofluorescence and electron microscopy. No obvious defects in cell morphology were observed by electron microscopy at 24 h post-induction (Supplementary Fig. 4), except in a small minority of cells (< 5%) which displayed axoneme defects and a swollen flagellar pocket (Supplementary Fig. 4C) compared to an estimated < 1% in uninduced cells.

The trypanosome flagellum is composed of a canonical 9 + 2 microtubular axoneme and the paraflagellar rod (PFR), a filamentous structure which runs parallel to the axoneme [43]. In order to analyse the effects of TbARL6 knockdown on the flagellum, the length of the PFR was measured in non-dividing cells (containing one nucleus and one kinetoplast, 1N1K) stained with DAPI and anti-PFR antibody prior to and following the induction of RNAi. The PFR extends to the distal tip of the flagellum in induced cells and therefore can be used as a reliable marker of flagellum length (Fig. 3C). Body length was also measured in non-dividing cells stained with DAPI and anti-α tubulin (Fig. 3D). There was a modest but highly significant decrease in PFR length at 24 and 48 h post-induction compared to wild-type and to uninduced cells (p = < 0.001) (Fig. 3E). Cells with shorter flagella remained motile, which is consistent with the observed normal growth described above. Motility is known to be essential for viability of this parasite stage [28] and any defects in this process would have resulted in cell death. We also observed a significant decrease in PFR length in uninduced RNAi cells compared to the parental line (p = < 0.001), which may be due to leaky expression of double-stranded RNA in the absence of tetracycline, as described in previous studies [44]. The mean PFR length was restored at later time points, again suggesting the emergence of escape mutants. Conversely, body length (including the region of the flagellum extending beyond the anterior tip of the body) was only marginally decreased in induced cells, with statistical significance at 48h compared to wild-type cells (p = < 0.01) (Fig. 3F). Our data therefore suggest that a moderate reduction in TbARL6 protein level resulted in a significant decrease in flagellum length but otherwise did not seem to affect cell growth or morphology.

We also tested the ability of BSF cells to establish an infection in a mouse in vivo model (data not shown). TbARL6 RNAi was induced with tetracycline for 4 h before infection and levels of parasitaemia checked at 48 and 72 h post-infection. Parasite concentrations in the blood were greater than 5 × 10^7^/ml in mice infected with either the parental or RNAi line by 72 h, indicating that parasites with a modest reduction in ARL6 due to RNAi retain the ability to mount an infection in vivo.

### 3.5 Attempts to make a TbARL6 knockout line

As RNAi knockdown was incomplete, we attempted to generate a BSF knockout line in which both alleles of the *ARL6* gene were replaced by antibiotic resistance selection markers. Parasite lines with one deleted allele were readily produced but no double knockout clones could be generated despite repeated transfections. Of a potential 192 clones (8 × 24 well plates) only two clones were successfully cultivated, a 50-fold reduction in efficiency compared to transfections to delete the first allele of the gene. Both of these clones encoded the two antibiotic resistance genes but also retained a copy of the *ARL6* gene, suggesting gene amplification events had occurred in these cells. These results are in direct contrast to those obtained in our recent study on a unrelated trypanosome gene *DIP13*. Both alleles of this gene were successfully deleted from the *T. brucei* genome using the same methods described here, with similar transfection efficiencies observed for the first and second transfections and no growth defect was found in *DIP13* null cells [45].

As an alternative approach, attempts were made to produce a conditional knockout line in which both alleles of the *ARL6* gene were deleted in cells expressing TbARL6^MYC^ under the control of a tetracycline-inducible promoter. As before, single allele deletion was achieved but no double knockout clones could be produced. Southern blotting and PCR showed that the TbARL6 locus was still present following correct integration of two antibiotic resistance cassettes in 8 independent clones (data not shown), again indicating that gene amplification had occurred in these parasites. Therefore, while moderate knockdown of the protein has no effect on cell growth, attempts to delete both alleles of the gene have so far been unsuccessful, resulting in either transfection failure or genetic rearrangements. When put into context with current dogma in kinetoplastid research, this suggests that the *TbARL6* gene could in fact be essential for viability and that the myc-tagged protein used for complementation (which localises differently in the cell) is not sufficient to rescue cells. Further studies are therefore required to determine whether TbARL6 is essential for viability in the parasite.

### 3.6 TbARL6 binds to tubulin

**Fig. 4:**
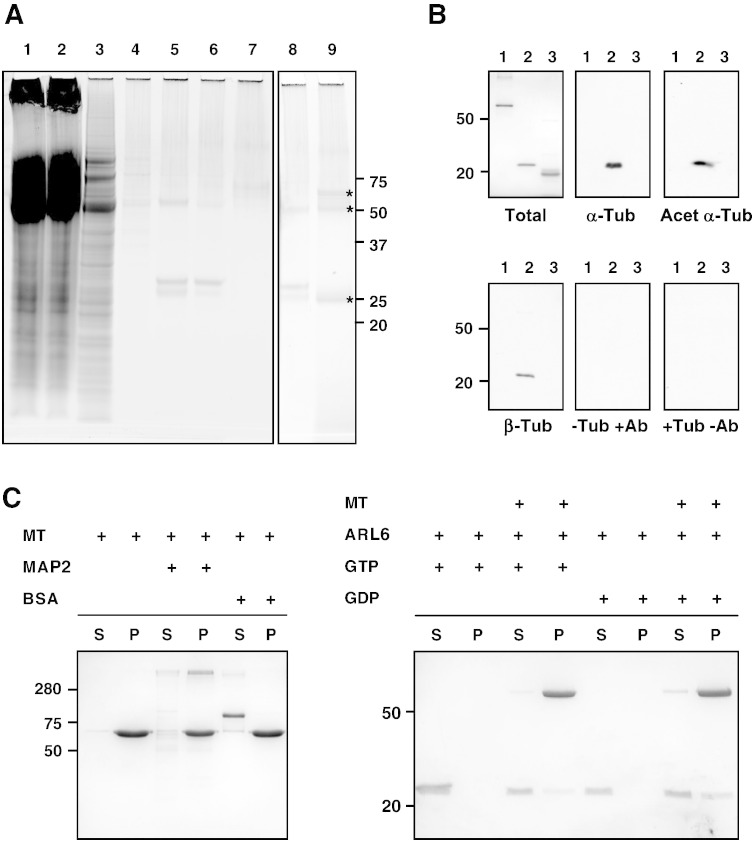
Identification of tubulin as an interacting partner of *T. brucei* ARL6. (A) PTP purification of TbARL6. Samples were separated by SDS-PAGE and stained with Coomassie. 1, Input material (0.05% of fraction loaded), 2, 3, 4, Flowthrough and washes of IgG Sepharose column (0.05%, 0.2%, and 0.13%), 5 and 8, TEV protease eluate (1%), 6, Flowthrough of the anti-ProtC matrix (0.3%), 7, Flowthrough of StrataClean resin (2.9%), 9, Final eluate (100%). The final eluted fraction contains three major protein bands (marked by asterisks) which were identified as protein C-tagged TbARL6 (23 kDa), α/β-tubulin (50 kDa) and a keratin contaminant (~ 65 kDa). (B) Tubulin overlay assay. Purified recombinant proteins (1 μg per lane) were separated by SDS-PAGE and either stained with Coomassie (Total) or transferred to PVDF (remaining panels). 1, Human Ras (R9894, Sigma), 2, TbARL6, 3, bovine serum albumin (New England Biolabs). Blots were incubated in tubulin solution overnight, then probed with mouse monoclonal antibody TAT1 against α-tubulin (α-Tub), monoclonal antibody clone 6-11B-1 which specifically recognises acetylated α-tubulin (Acet α-Tub) or monoclonal antibody TUB 2.1 against β-tubulin (β-Tub). Control immunoblots were processed with TAT1 antibody but without tubulin (− Tub + Ab) or without primary antibody (+ Tub − Ab). (C) Microtubule binding protein assay. Purified proteins were incubated with a microtubule preparation before ultracentrifugation. Soluble (S) and pellet (P) fractions were subjected to SDS-polyacrylamide electrophoresis and stained with Coomassie. MT, microtubules. MAP2, Microtubule associated protein 2 (280 kDa, positive control), BSA, bovine serum albumin (68 kDa, negative control). Corresponding protein marker positions are shown (kDa).

In order to find the binding partners of TbARL6, we used a modified version of tandem affinity purification (TAP), which involves fusion of the protein to Protein A and Protein C epitopes (termed a PTP tag) [32] to enable highly-specific purification of protein complexes from crude cell lysate. A construct encoding C-terminal PTP-tagged ARL6 was inserted into the endogenous ARL6 locus of *T. brucei* procyclic parasites to produce cells expressing tagged protein at endogenous levels. This reduced the possibility of erroneous interactions due to overexpression but necessitated the use of large volumes of cells in order to produce detectable amounts of final product. Following a series of affinity purification steps, three proteins were detected in the final eluate (Fig. 4A). These were identified by mass spectrometry as TbARL6, α/β-tubulin and a keratin contaminant, with identical results produced in four independent biological replicates. As tubulin is highly abundant in trypanosomes and often found as a contaminant in protein interaction studies, additional studies were required to validate this protein as a binding partner of TbARL6.

This was investigated further using a tubulin overlay assay (Fig. 4B). Immunoblots of N-terminal His-tagged recombinant ARL6 (which cannot be *N*-myristoylated) and controls were incubated in porcine tubulin solution before probing with anti-tubulin antibodies. The presence of a 22 kDa band in the ^His^ARL6 lane (Fig. 4B) clearly showed binding of this protein to α-tubulin *in vitro*, whereas no signal was produced in lanes containing either BSA or the small GTPase Ras. The same band was also detected when blots were probed with antibodies specific for acetylated α-tubulin and for β-tubulin. No bands were visible in the absence of tubulin or primary antibody (Fig. 4B).

To further validate our findings, an *in vitro* microtubule binding assay was performed in which purified proteins were incubated with a preparation of polymerised tubulin prior to ultracentrifugation (Fig. 4C). N-terminal His-tagged recombinant ARL6 was completely soluble in the absence of microtubules but following incubation with a microtubule preparation in the presence of guanine nucleotide (Fig. 4C, right hand panel), a proportion of the TbARL6 protein relocated to the insoluble fraction (36% of the protein in the presence of GDP, 20% with GTP). Control lanes show that a majority of MAP2 was found in the insoluble fraction in the presence of microtubules, whereas BSA which does not bind microtubules was detected exclusively in the soluble fraction (Fig. 4C, left hand panel).

To summarise these findings, our data indicate that TbARL6 is a tubulin-binding protein (with evidence from a range of techniques) and has the ability to bind both unpolymerised tubulin and assembled microtubules (albeit transiently or at lower affinity than the MAP2 positive control). Further, these interactions are not dependent on *N*-myristoylation or on specific nucleotide binding conformation of the small GTPase.

We also tested the ability of TbARL6 to alter the *in vitro* polymerisation of tubulin in the presence of taxol, monitoring the increase in turbidity as microtubules are produced, as described previously [34]. No significant differences were seen in the presence or absence of recombinant TbARL6 (data not shown). Thus, while our data strongly suggest interaction between TbARL6 and tubulin, the functional relevance of this interaction in vivo will require further investigation.

### 3.7 TbARL6 nucleotide interactions

**Fig. 5:**
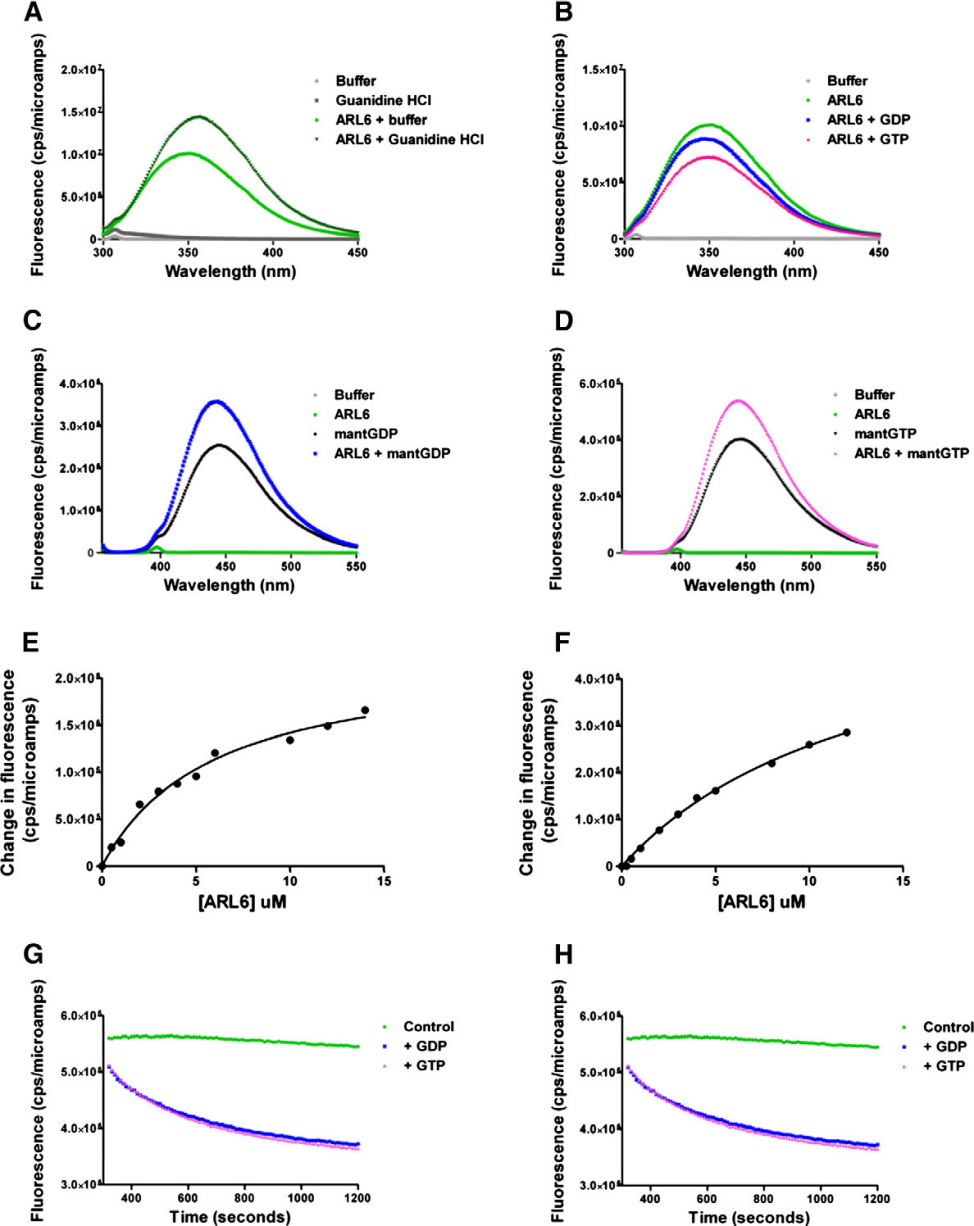
*T. brucei* ARL6 nucleotide interactions. Purified recombinant TbARL6 was used at 5 μM in 50 mM phosphate buffer unless otherwise stated. (A) Tryptophan fluorescence emission spectra of TbARL6 in the presence and absence of 6 M guanidine hydrochloride. (B) Tryptophan fluorescence emission spectra of TbARL6 in phosphate buffer and 10 mM MgCl_2_ in the presence and absence of 12.5 μM unlabelled GDP or GTP. (C, D) Mant fluorescence emission spectra of mantGDP (C) and mantGTP (D) in the presence and absence of TbARL6 protein. (E, F) Dissociation constants (Kd) for TbARL6 and mantGDP (E) or mantGTP (F) were calculated by non-linear regression (one site specific binding) from data obtained at a single emission wavelength (GDP, 445 nm, GTP, 447 nm) with a fixed concentration of nucleotide and a range of protein concentrations. (G, H) Guanine nucleotide exchange by TbARL6. Protein was prebound to mantGDP (G) or mantGTP (H), then mixed at 300 s with a large excess of unlabelled nucleotide. The decrease in fluorescence was recorded over time and dissociation rate constants (κdiss) calculated by non-linear regression (one phase exponential decay). Controls represent protein and mant-labelled nucleotide without the addition of unlabelled nucleotide. All data are representative of at least 3 independent experiments.

In addition to identifying protein binding partners of TbARL6, we studied the nucleotide interactions of recombinant protein. To test the folding integrity of purified protein, emission spectra were recorded for TbARL6‐^His^ either in phosphate buffer or following denaturation in 6 M guanidinium hydrochloride. Denaturation caused an increase in tryptophan fluorescence and a red shift of the emission maximum from 350 nm to 356 nm (Fig. 5A). This suggests that the protein has a hydrophobic core and is capable of folding in phosphate buffer in the absence of Mg^2+^ or nucleotide. TbARL6 is therefore likely to be stable in a nucleotide-free state. Intensity of the intrinsic fluorescence of TbARL6‐^His^ decreased in the presence of GDP/GTP and 10 mM MgCl_2_ (Fig. 5B), with GTP exerting the greatest effect. No change in fluorescence was seen in the absence of magnesium (data not shown) confirming that nucleotide binding, as for other Arf GTPases, is Mg^2+^‐dependent.

TbARL6‐^His^ showed rapid binding with mant-labelled GDP and GTP, causing an increase in the fluorescence intensity of the nucleotides (Fig. 5C and D). Fluorescence reached a plateau within 3 min (data not shown) and remained relatively constant when measured at 10 s intervals for 20 min (control samples in Fig. 5G and H). Equilibrium binding constants were determined by titrating mant-labelled nucleotides with increasing concentrations of protein and plotting the change in fluorescence (Fig. 5E and F). The dissociation constant (Kd) value of each interaction was calculated by non-linear regression (one site specific binding) and compared with known values for other small GTPases (Table 1) [46–52]. The Kd of TbARL6-^His^ with GDP is 4.35 μM (± 1.12) and with GTP is 13.71 μM (± 3.52). We attempted to corroborate these data with unlabelled nucleotides by isothermal titration calorimetry (ITC) but the protein precipitated in response to the vigorous agitation required.

Dissociation rate constants (κdiss) were measured by loading TbARL6‐^His^ with mant-labelled nucleotide, then adding a 100-fold excess of unlabelled nucleotide and recording the decrease in fluorescence as the mant-labelled molecules were displaced (Fig. 5G and H). For both GDP and GTP, the resultant curves fitted one-phase rather than two-phase exponential decay, indicating a single dissociation event. Similar values were produced when displacing the mant nucleotides with either GDP or GTP (Fig. 5G and H and Table 1). The κdiss for mantGDP was found to be 0.0076 s^− 1^ (± 0.0014) when displaced with unlabelled GDP and 0.0093 s^− 1^ (± 0.0013) when displaced with unlabelled GTP. The equivalent values for mantGTP were 0.0047 (± 0.0006) with GDP and 0.0041 (± 0.0007) with GTP.

### 3.8 Interaction between TbARL6 and the BBSome

**Fig. 6:**
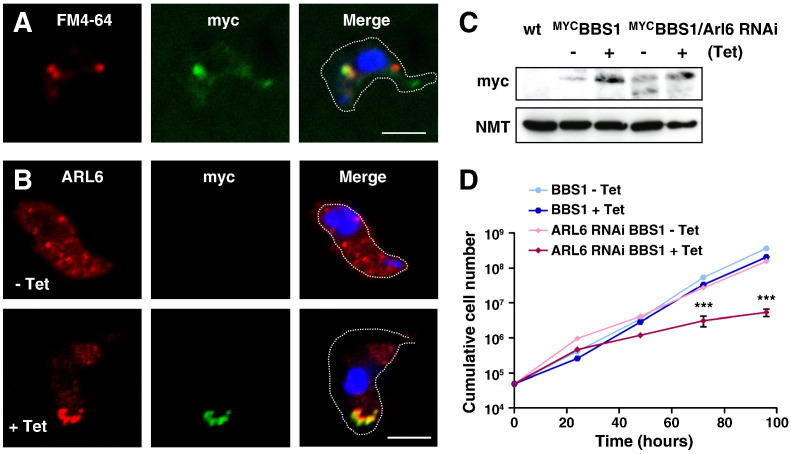
Association of TbARL6 with the BBSome. (A) Immunofluorescence analysis of *T. brucei* BSF transfected line 427/pMYCBBS1 grown in the presence of tetracycline for 24 h. Cells were incubated on ice with the lipophilic dye FM4-64 (red) to stain the flagellar pocket prior to fixing, then probed with mouse anti-myc (green) and co-stained with DAPI (blue). Bar, 5 μm. (B) Cells as in (A) above were grown in the absence (− Tet) or presence (+ Tet) of tetracycline for 72 h, then probed with mouse anti-myc (green) and rabbit anti-ARL6 (red) and co-stained with DAPI. Bar, 5 μm. (C) Total cell lysates (1 × 10^7^ cells/lane) from BSF parental line Lister 427 (wt) and cell lines 427/pMYCBBS1 and 427/p2T7ARL6/pMYCBBS1 grown in the absence (−) or presence (+) of tetracycline for 24 h were immunoblotted and probed with mouse anti-myc and anti-NMT to monitor equal sample loading. (D) Cumulative growth of BSF transfected lines 427/pMYCBBS1 (BBS1) and 427/p2T7ARL6/pMYCBBS1 (BBS1 Arl6 RNAi) in the absence (−) and presence (+) of tetracycline, monitored over a 5 day time course (mean ± SD shown, n = 3) with statistical analysis by 1 way ANOVA (*** = p < 0.001).

Human Arl6 is now believed to interact with the BBSome complex by directly binding to the BBS1 subunit [11,53]. To investigate whether this interaction is conserved in kinetoplastids, an N-terminal myc tagged form of the *T. brucei* orthologue of BBS1 was inducibly expressed in bloodstream form parasites, as confirmed by immunofluorescence (Fig. 6A–B) and immunoblotting (Fig. 6C). By 24 h post-induction of ^MYC^BBS1 expression, tagged protein was detected in a dense focus at the posterior end of the cell. Co-localisation with the lipophilic dye FM4-64 identified this as the flagellar pocket (Fig. 6A). A striking difference in endogenous TbARL6 protein localisation can be seen following the induction of BBS1 overexpression, with translocation from a punctate pattern to a densely stained region that co-localises with the ^MYC^BBS1 protein at the posterior end of the parasite (Fig. 6B). Induction of ^MYC^BBS1 expression had little effect on parasite division until 72 and 96 h post-induction when a significant reduction in cell growth was detected compared to uninduced cells (p = < 0.01). When ^MYC^BBS1 was expressed in the TbARL6 RNAi cell line, there was a synergistic effect with a highly significant decrease in growth by 72 and 96 h post-induction compared to uninduced cells, induction of TbARL6 RNAi alone or ^MYC^BBS1 expression alone (p = < 0.001 in all cases) and the presence of rounded cells (data not shown). These results strongly suggest that the functional link between ARL6 and BBS1 observed in human cells is conserved in trypanosomes.

## 4 Discussion

### 4.1 Interactions of TbARL6

In this study, we show that the orthologue of Arl6 in trypanosomes is *N*-myristoylated (unlike other Arl6 orthologues), is associated with small vesicles throughout the parasite body and can associate with tubulin. Knockdown of TbARL6 expression leads to a significant decrease in flagellum length but no motility defect. During the course of these studies, a report was published describing the interaction between human Arl6 and the BBSome [11]. We were unable to detect components of the BBSome in our affinity purification experiments (Fig. 4A) but this was not surprising given that human Arl6 was not co-purified with the other components of the BBSome in the original study identifying this complex [12]. Instead, a GTP-locked Arl6 mutant was later shown to bind to the BBSome [11] suggesting that binding of the GTPase to these components may be transient and dependent on nucleotide binding status. As TbARL6 does not contain the equivalent of the highly conserved Gln 71 (discussed in Section 4.6), further investigation of relevant residues will be required before analysis of a GTP-locked mutant is feasible. A recent study suggested that the interaction between Arl6 and BBS1 may be regulated by the Arl6 N-terminus [53]. Further studies are required to determine if *N*-myristoylated TbARL6 retains the ability to bind BBS1. Moreover, the full complement of Arl6 and TbARL6 interacting proteins and their roles in protein trafficking remain to be fully characterised. An earlier study used yeast two-hybrid screening to identify six interacting partners of human Arl6, including SEC61beta, a subunit of the heterotrimeric protein conducting channel SEC61p and a molecule of unknown function, Arl6 interacting protein 1 (Arl6ip) [54]. However, there are no clear orthologues of any of these interacting proteins in trypanosomes that are identifiable by computational analysis.

### 4.2 Loss of function of proteins associated with Bardet–Biedl syndrome

A range of model systems have been used to study loss of function of the Bardet–Biedl associated proteins. With few exceptions, disruption of cilium function is the consistent phenotype regardless of which BBS gene is targeted, leading to specific trafficking or signalling defects and downstream events depending on the model organism studied. Unlike many of the IFT components, loss of BBS gene function does not appear to be lethal [18,55]. Also, the assembly of cilia/flagella is still able to occur in the absence of specific subunits of the BBSome complex in all eukaryotic systems studied to date, with the notable exception of flagellum production in the spermatozoa of knockout mice [18,53,56–60]. Abnormalities in rhodopsin trafficking in BBS knockout mice leads to degeneration of photoreceptor cells [56,61,62], a phenotype which can be prevented in *Bbs4* knockout mice by adenoviral delivery of the *Bbs4* gene [63]. *Bbs3* (Arl6) knockout mice display many of the characteristic features of Bardet–Biedl syndrome, including retinal degeneration and male infertility but also have unique phenotypes such as severe hydrocephalus, suggesting that the protein may have roles in addition to BBSome recruitment [53]. Knockdown of BBS genes (including *BBS3*) in zebrafish causes delayed retrograde melanosome transport and disruption of the Kupffer's Vesicle, a fluid-filled ciliated epithelial sac found in embryonic fish [58–60]. Changes in Sonic Hedgehog (shh) expression are also observed, leading to alterations in fin bud patterning [59]. Conversely, specific knockdown of an alternative transcript of *BBS3* termed BBS3L does not produce this phenotype, instead resulting in retinal defects [64], therefore suggesting that these two ARL6 variants have distinct non-overlapping functions. BBSome insertional mutants in *C. reinhardtii* can assemble normal length motile flagella and have normal IFT particle structure and movement but display a defect in phototaxis. This is due to an intraflagellar accumulation of four putative signalling proteins, indicating that the BBSome has a role in export of signalling proteins from the flagellum [18].

In trypanosomes, we found that a modest knockdown of ARL6 does not kill the parasite but causes a significant decrease in flagellum length, suggesting some evolutionary conservation in the function of this protein. Flagellum motility and maintenance of flagellar pocket integrity are essential for viability of the host bloodstream form (BSF) of the parasite and disruption of these by RNA interference invariably leads to cell death [28,65,66]. Partial knockdown of TbARL6 expression did not cause widespread cell death, unlike RNAi knockdown of the related small GTPases ARL1 and ARF1 in *T. brucei* [21,22]. Nevertheless, despite repeated attempts, we were unable to generate a double knockout null line and further studies will be required to confirm the full effects of ARL6 loss of function in this organism. Further, we found that overexpressed BBS1 in *T. brucei* localises to the flagellar pocket and results in a translocation of endogenous TbARL6 to this site. A combination of BBS1 overexpression and knockdown of ARL6 has a synergistic inhibitory effect on cell growth, indicating a close link between ARL6 and the BBSome in trypanosomes.

### 4.3 The BBSome and Rab8

BBS1 is able to directly bind Rabin8 [12] and a model has been proposed in which the BBSome may be able to regulate activation of the multifunctional small GTPase Rab8, leading to the sorting and transport of cargo to the cilium [12,67] and potentially across the periciliary barrier for conveyance to the IFT system [11]. However, while Rab8 is found in almost all eukaryotes [68], the BBSome is restricted to ciliated/flagellated organisms [9]. Moreover, flagellated protozoan parasites have conserved BBSome subunits but lack a member of the Rab8/Rab10/Rab13 clade [9,69], indicating that there is only a partial overlap in functional mechanism between the BBSome and Rab8, and that Rab8-independent BBSome processes must exist, at least in protozoa.

### 4.4 Localisation of ARL6 and the BBSome

Human Arl6 localises to the distal end of basal bodies and to primary cilium membranes [11,70] and the orthologue of this protein has been detected in the flagellar proteome of *C. reinhardtii* [71] but not in similar studies of *T. brucei* procyclic and BSF parasites [28,45]. Human BBSome components have been detected in two pools, localising to centriolar satellites and inside the primary cilium. Affinity purification techniques have shown that the subunit BBS4 interacts directly with PCM1, the core component of the centriolar satellites, which may act as a chaperone to prevent coat assembly before it is required. In contrast, the BBSome found within the primary cilium is dissociated from PCM1 and associates instead with GTP-bound Arl6 [11,12]. PCM1 and centriolar satellites are not conserved in protozoans, therefore a different mechanism may be required for safe transport of the BBSome components from their site of synthesis (as yet unidentified) to the flagellar pocket or other locations in the cell. Our data suggest that endogenous TbARL6 is associated with small vesicles throughout the parasite body and we speculate that TbARL6 could mediate delivery of the BBSome components on these vesicles.

### 4.5 TbARL6 localisation and tubulin association

Our protein interaction studies identified tubulin as a potential binding partner of TbARL6, independent of *N*-myristoylation or nucleotide binding status. This suggests a possible association of ARL6-positive vesicles with cytoplasmic microtubules to facilitate transport to the flagellar pocket, although these structures have not yet been visualised in *T. brucei* [72]. No significant co-localisation was found between endogenous TbARL6 and the endosomes but myc-tagged ARL6 partially co-localises with the early endosomal marker Rab5 in parasites, signifying either mislocalisation due to the epitope tag or abnormal accumulation of overexpressed protein at this stage due to saturation of the transport mechanism. Although the N-terminus is known to be critical for *N*-myristoylated Arf/Arl function, blocking the C-terminus by epitope-tagging may also be detrimental to protein function [27,73]. It is interesting to note that the G2A mutant of TbARL6 shows a subcellular localisation consistent with binding to the subpellicular microtubules at the plasma membrane. We hypothesise that *N*-myristoylation could provide the switch required for additional interactions to occur which limit potential association with tubulin and thus enable TbARL6 to reach its correct vesicular localisation. In the absence of this modification, TbARL6 may instead accumulate at a “default” localisation in the region with the highest tubulin concentration, that of the subpellicular cortex underlying the plasma membrane [74]. Endogenous TbARL6 has not been detected in detergent-insoluble cell extracts (which contain the cytoskeleton), showing that any association with microtubules is probably transient. An intriguing alternative hypothesis to direct microtubule binding is that tubulin may be a cargo molecule of ARL6-positive vesicles. It has been hypothesised that all proteins destined for the ciliary membrane and axoneme, including tubulin, may be transported via association with Golgi-derived vesicles which exocytose with the basal body and then the ciliary membrane [75]. Tubulin has been identified in vesicles destined for the cilium and may in fact peripherally associate with the outside of vesicles prior to exocytosis with the ciliary membrane [75], in a similar mechanism to that described for F-actin delivery on endosome-derived vesicles to ingressing cleavage furrows during cytokinesis [76].

Two other proteins in the Arf family, Arl7 and Gie/Arl8, have been shown to date to bind directly with tubulin [77,78]. Arl7 binds to α-tubulin [77], shows upregulated expression in response to cholesterol loading and has been implicated in transport of apolipoprotein AI-dependent cholesterol export [77,79]. Gie/Arl8 is essential for equal segregation of chromosomes in mammalian cells and *Drosophila* [78]. Like *T. brucei* ARL6, Gie is able to bind to both α- and β-tubulin, independent of the nucleotide-binding status of the GTPase [78]. The ability to bind tubulin is retained in mutant forms of Gie1 (Arl8B) lacking the Switch I or Switch II effector binding domains, suggesting that tubulin is a binding partner rather than an effector or regulator molecule. Other members of the Arf family, Arl2 and Arl3, have roles in microtubule-related processes but these are not believed to require a direct interaction with tubulin. GDP-bound Arl2 binds to tubulin cofactor D, preventing the disassembly of microtubules [2]. The orthologue of Arl2 in *T. brucei* is required for cleavage furrow ingression during cytokinesis and knockdown causes the production of multinucleated cells [27]. Arl3 is closely related to Arl2 and is able to bind all Arl2 effector proteins with the exception of cofactor D [80]. Arl3 has an additional set of interacting partners, human retinal gene 4 (HRG4/UNC119) and retinitis pigmentosa 2 (RP2), with which it can form a ternary complex [81]. Similar to Arl6, Arl3 has been detected on primary cilia in human photoreceptor cells [82,83] and Arl3 knockout mice develop photoreceptor degeneration [84]. Kinetoplastids have three putative Arl3 orthologues [22], of which one, termed ARL-3A, has been studied in detail. Disruption of ARL-3A function in *Leishmania* by expression of either GTP-locked or GDP-locked forms and in *T. brucei* by RNAi knockdown causes significant shortening of flagella without loss of motility [85–87], as found here with knockdown of *T. brucei* ARL6. ARL-3A in *Leishmania amazonensis* is found in a punctate pattern [87] and may therefore have a vesicular localisation similar to that for TbARL6. Recent studies have shown that Arf proteins can act in synergy to produce an activation cascade, as shown previously for Rabs [88], with a minor population of GTP-bound activated Arf6 or Arl4 binding to the PH domain of the Arno GEF to enhance binding of Arf1 to the Sec7 domain, thus triggering Arf1 activation [89]. Our data suggest that *T. brucei* ARL6 has spatial and functional similarities to ARL-3A and there may conceivably be an association between the two proteins, either in function or in triggering activation.

### 4.6 Guanine nucleotide binding properties of TbARL6

As a small GTPase, the nucleotide binding properties of Arl6 will almost certainly be critical for its function. As a reflection of this, many of the Arl6 mutations described in BBS patients are missense mutations of the highly conserved amino acids required for guanine nucleotide association [90]. Expression of GDP- and GTP-locked variants of Arl6 in a human cell line does not alter cell growth or gross morphology but significantly changes the proportion of cells possessing cilia and the length of assembled cilia. Overexpression of wild-type Arl6 causes an increase in Wnt signalling, as measured by the level of β-catenin transcriptional activity, but expression of nucleotide-locked variants of Arl6 has no effect on this response [70]. The amino acid residues Thr 31 and Gln 71 (Arf1 numbering) are highly conserved in nearly all Ras-like small GTPases, with critical roles in GTP binding and hydrolysis, respectively. Sequence alignment of putative Arl6 orthologues (Supplementary Fig. 5) shows that while Thr 31 is conserved in the kinetoplastid orthologues (except in *T. cruzi*), the equivalent residue of Gln 71 in Arf1, is substituted by alanine in the parasite Arl6 sequences (residue 63). Gln 71 is also absent from the larger GTPase dynamin, which has the ability to self-assemble into higher order structures, resulting in active-site conformational changes which in turn stimulate GTP hydrolysis [91].

In light of this information, we investigated the ability of recombinant His-tagged TbARL6 to bind nucleotides using tryptophan and fluorescence spectroscopy. There are two tryptophan residues in TbARL6, one of which resides in the Switch II effector binding domain. Our data indicate that nucleotide binding causes a local conformational change in this region which partially quenches fluorescence, as shown for Rab5 [92]. Previous studies have shown that Arf1 requires the presence of lipids and Mg^2+^ for nucleotide binding and is unstable *in vitro* in a nucleotide-free state. Arf1 has a very high affinity for guanine nucleotides (nanomolar range) and the exchange rate is extremely slow [48]. In contrast, the related protein Arl2 is stable in a nucleotide-free state and has low affinity for nucleotides due partly to a rapid exchange rate [46]. We conclude from our data that TbARL6-^His^ protein is stable in the absence of nucleotides and is able to bind guanine nucleotides with low affinity in the absence of lipids, with relatively rapid exchange rates. Therefore, the protein appears to be similar in terms of nucleotide binding characteristics to Arl2. Both TbARL6 and dynamin lack the equivalent residue of Gln 71 in Arf1, an amino acid shown to be crucial for GTP hydrolysis. As both of these proteins also exhibit low affinity for GTP, it is possible that this residue may have a role in modulating nucleotide affinity in small GTPases. Alternatively, the His tag may exert some inhibitory effects or TbARL6 may require other factors such as membrane lipids for optimal nucleotide association.

### 4.7 Concluding remarks

In conclusion, we have investigated the functions of the Arl6 orthologue in *T. brucei*, a divergent lower eukaryote which encodes the BBSome components but lacks a Rab8 orthologue. It is yet to be proven that the BBSome has a role in transporting cargo to the flagellum in kinetoplastid parasites but the functional analysis of *T. brucei* ARL6 presented here suggests that this mechanism is fundamentally conserved and required for flagellum extension. We propose that there is a conserved functional association between the BBSome and ARL6 in *T. brucei* which is required for flagellar extension in a Rab8 null background. Remarkable recent evidence has shown that the scope of the intraflagellar transport system is not restricted to that of transport within the flagellum. One of the IFT components, IFT20, has a dynamic localisation in photoreceptor cells, moving between the Golgi and the primary cilium, therefore providing a potential transport link between the endomembrane system and the IFT machinery [93]. Considering the widespread distribution of ARL6 in trypanosomes, we cannot rule out the possibility that this protein may also have a role linking transport between multiple sites in the parasite. Further studies will be required to elucidate the exact molecular mechanisms of ARL6 and related BBSome functions in trypanosomes and other eukaryotic cells.

The following are the supplementary data related to this article.

Supplementary Fig. 1 Expression of recombinant TbARL6 and production of TbARL6 antibody. (A) *E. coli* strain BL21 star was transformed with the construct pET-^His^ARL6 and expression of recombinant protein induced with 1 mM IPTG for 4 h at 30 °C. Total lysates were prepared using cell pellets from 1 ml aliquots of culture, which were resuspended in 100 μl ice-cold PBS and sonicated (3 × 10 s). Soluble and insoluble fractions were collected following centrifugation at 4 °C at 16,000 *g* for 30 min. Cell fractions were separated by SDS-PAGE and stained with Coomassie, with the equivalent of 100 μl of cell culture loaded per lane in 1× Laemmli buffer. Un, total lysate from uninduced cells. T, total lysate from cells induced with IPTG for 4 h. S, soluble fraction of induced cells. P, insoluble fraction of induced cells. (B) Immunoblots of *T. brucei* PCF total cell lysate (1 × 10^7^ cells/lane) probed with anti-TbARL6 (1:500 dilution) prior to (Ab1) and following (Ab2) affinity purification of the antibody. Corresponding protein marker positions are shown (kDa).

Supplementary Fig. 2 Localisation of endogenous and mutant forms of *T. brucei* ARL6. (A) Immunofluorescence analysis of *T. brucei* bloodstream form (BSF) line Lister 427 pre-treated with Alexa Fluor488 conjugated ConA (green) at 15 °C to stain the flagellar pocket and early endosomes, prior to fixing. Cells were then probed with anti-TbARL6 (red) and co-stained with DAPI (blue). (B) Immunofluorescence analysis of BSF transfected lines 427/pTbARL6MYC, 427/pTbARL6-G2AMYC and 427/pTbARL6-T21NMYC grown in the presence of tetracycline for 24 h. Cells were probed with mouse anti-myc (green) and rabbit anti-TbRab5 (red) and co-stained with DAPI (blue). Bar, 5 μm. (C) Total cell lysates (5 × 106 cells/lane) from BSF parental line Lister 427 (wt) and BSF transfected lines 427/pTbARL6MYC, 427/pTbARL6-G2AMYC and 427/pTbARL6-T21NMYC grown in the absence (−) or presence (+) of tetracycline for 24 h were immunoblotted and probed with mouse anti-myc, anti-TbARL6 and anti-NMT to monitor equal sample loading.

Supplementary Fig. 3 Transmission immuno-electron micrographs of *T. brucei* BSF parasites. (A) Parental line Lister 427 probed with 10 nm colloidal gold conjugated goat-anti-rabbit only as a negative control for the data shown in Fig. 1. (B–D) BSF transfected line 427/pTbARL6MYC probed with mouse anti-myc (C–D) or no primary antibody control (B) followed by detection with 10 nm colloidal gold conjugated goat-anti-mouse. Image D is an enlarged view (× 3.1) of the boxed area in image C. BB, basal body. Fl, flagellum. FP, flagellar pocket. Bar, 200 nm (A, B, D) or 1 μm (C).

Supplementary Fig. 4 Transmission electron micrographs of cell line 427/p2T7ARL6 (RNAi) grown in the presence of tetracycline for 24 h. Fl, flagellum. FP, flagellar pocket. FPC, flagellar pocket collar. BB, basal body. SMT, subpellicular microtubules. Ac, acidocalcisome. Bar, 500 nm.

Supplementary Fig. 5 Alignment of kinetoplastid ARL6 orthologues and related protein sequences. Grey shaded areas indicate Switch I and II effector domains. The amino acid residues corresponding to Thr 31 and Gln 71 (human Arf1 numbering) are shown in yellow shading. Sequence accession numbers (GenBank/TriTrypDB): *Trypanosoma brucei*, Tb927.8.5060; *Trypanosoma cruzi*, Tc00.1047053508839.60; *Leishmania major*, LmjF16.1380; Human, AAH24239; Orangutan, NP_001127054.1; Dog, E2RRS0-CANFA; Mouse, AAC62194; Rat, AA162029; *Danio rerio*, AAI65137; *C. elegans*, CAA86319; *T. brucei* ARF1 (TbARF1), Tb09.211.4480; Human Arf1, P84077.2. Human–Long represents the long isoform of Arl6 described in Pretorius et al. (2010).

Supplementary Table 1 List of primer sequences.

## Figures and Tables

**Table 1:** Guanine nucleotide binding and exchange kinetics of TbARL6. Dissociation constants (Kd) and dissociation rate constants (κdiss) for the binding of N-terminal His-tagged *T. brucei* ARL6 to mant guanine nucleotides (mGDP and mGTP), compared to values obtained previously for other small GTPases using either mant or radioactive nucleotides (shown in separate columns). References: [1] M. Hanzal-Bayer, M. Linari, A. Wittinghofer, Properties of the interaction of Arf-like protein 2 with PDEdelta, J Mol Biol, 350 (2005) 1074–1082. [2] M. Linari, M. Hanzal-Bayer, J. Becker, The delta subunit of rod specific cyclic GMP phosphodiesterase, PDE delta, interacts with the Arf-like protein Arl3 in a GTP specific manner, FEBS Lett, 458 (1999) 55–59. [3] R.A. Kahn, A.G. Gilman, The protein cofactor necessary for ADP-ribosylation of Gs by cholera toxin is itself a GTP binding protein, J Biol Chem, 261 (1986) 7906–7911. [4] D.D. Binns, B. Barylko, N. Grichine, M.A. Atkinson, M.K. Helms, D.M. Jameson, J.F. Eccleston, J.P. Albanesi, Correlation between self-association modes and GTPase activation of dynamin, J Protein Chem, 18 (1999) 277–290. [5] E.S. Burstein, I.G. Macara, Interactions of the ras-like protein p25rab3A with Mg2+ and guanine nucleotides, Biochem J, 282 ( Pt 2) (1992) 387–392. [6] B. Zhang, Y. Zhang, Z. Wang, Y. Zheng, The role of Mg2+ cofactor in the guanine nucleotide exchange and GTP hydrolysis reactions of Rho family GTP-binding proteins, J Biol Chem, 275 (2000) 25299–25307. [7] T. Terui, R.A. Kahn, P.A. Randazzo, Effects of acid phospholipids on nucleotide exchange properties of ADP-ribosylation factor 1. Evidence for specific interaction with phosphatidylinositol 4,5-bisphosphate, J Biol Chem, 269 (1994) 28130–28135.

	mGDP	mGTP	GDP	GTP	GTPγS	Ref.
Kd (μM)						
TbARL6	4.354	13.710				
Arl2	0.492	0.173				[1]
Arl3	0.240	48.000				[2]
Arf1			0.040	0.090	0.020	[3]
Dynamin	100.000	2.500				[4]
Rab3A			0.063		0.046	[5]
Cdc42	0.220		0.590		0.170	[6]
RhoA	0.110		0.480		0.160	[6]
Rac1	0.240		0.620		0.240	[6]
κdiss (s^− 1^)						
TbARL6	0.00757	0.00407				
Arl2	0.02679	0.00716				[1]
Arl3	0.00017	0.03510				[2]
Arf1			0.00035		0.00170	[7]
Dynamin	93.00000	2.10000				[4]
Rab3A			0.00018		0.00040	[5]
Cdc42			0.01700		0.01500	[6]
RhoA			0.01500		0.01000	[6]
Rac1			0.02900		0.03100	[6]
