# Supplementary figures and images for: A role for the vesicle-associated tubulin binding protein ARL6 (BBS3) in flagellum extension in Trypanosoma brucei
Source: Biochim Biophys Acta. 2012 Jul;1823(7):1178–91. doi: 10.1016/j.bbamcr.2012.05.007 (PMC3793860; doi:10.1016/j.bbamcr.2012.05.007)

## Slide 1
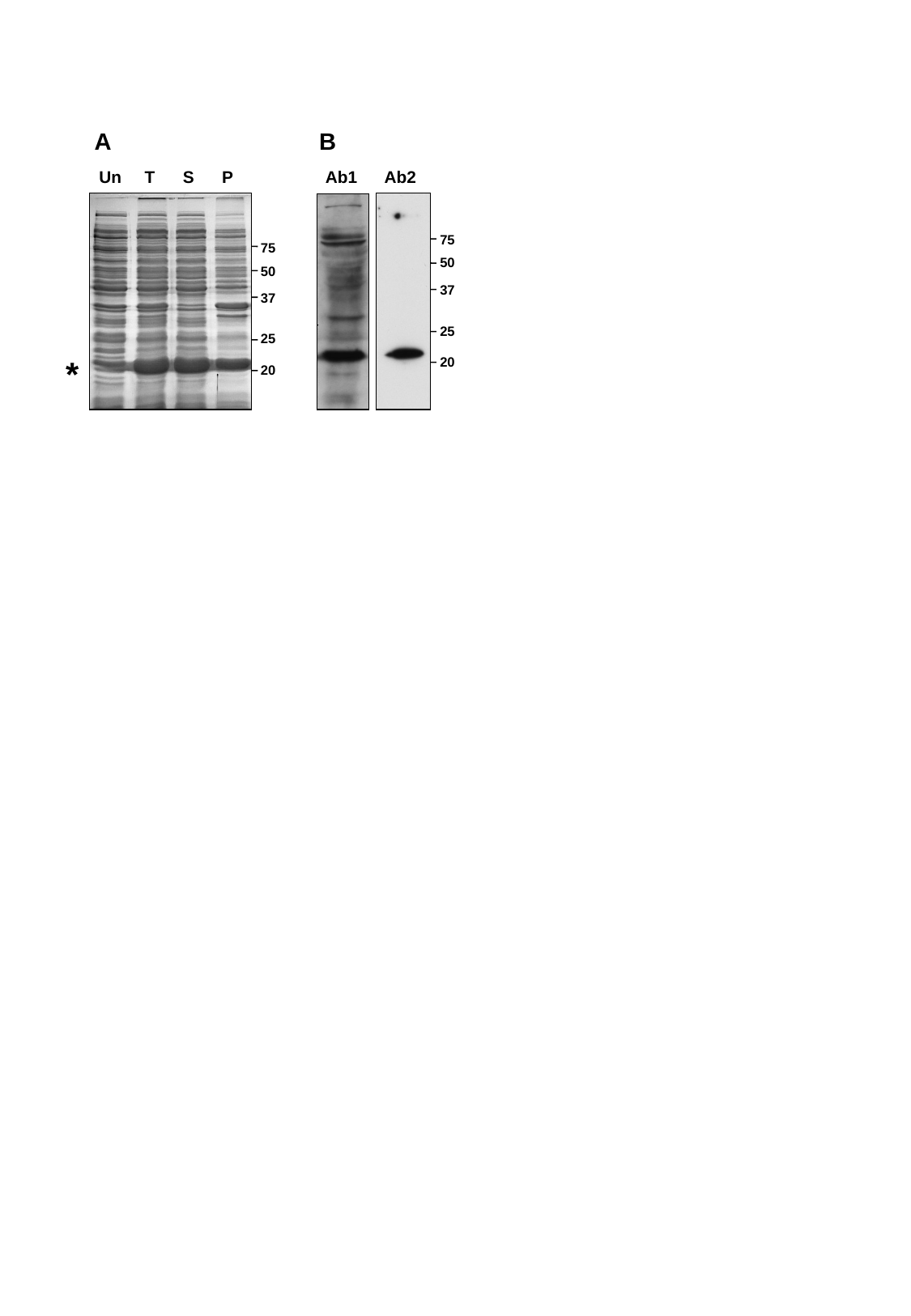

A
 B
 Un T S P Ab1 Ab2
75
50
37
25
20
*
75
50
37
25
20

Supplement: Supplementary Fig. 1 — Expression of recombinant TbARL6 and production of TbARL6 antibody. (A) E. coli strain BL21 star was transformed with the construct pET-HisARL6 and expression of recombinant protein induced with 1 mM IPTG for 4 h at 30 °C. Total lysates were prepared using cell pellets from 1 ml aliquots of culture, which were resuspended in 100 μl ice-cold PBS and sonicated (3 × 10 s). Soluble and insoluble fractions were collected following centrifugation at 4 °C at 16,000 g for 30 min. Cell fractions were separated by SDS-PAGE and stained with Coomassie, with the equivalent of 100 μl of cell culture loaded per lane in 1× Laemmli buffer. Un, total lysate from uninduced cells. T, total lysate from cells induced with IPTG for 4 h. S, soluble fraction of induced cells. P, insoluble fraction of induced cells. (B) Immunoblots of T. brucei PCF total cell lysate (1 × 10^7^ cells/lane) probed with anti-TbARL6 (1:500 dilution) prior to (Ab1) and following (Ab2) affinity purification of the antibody. Corresponding protein marker positions are shown (kDa). [file mmc1.ppt]

## Slide 1
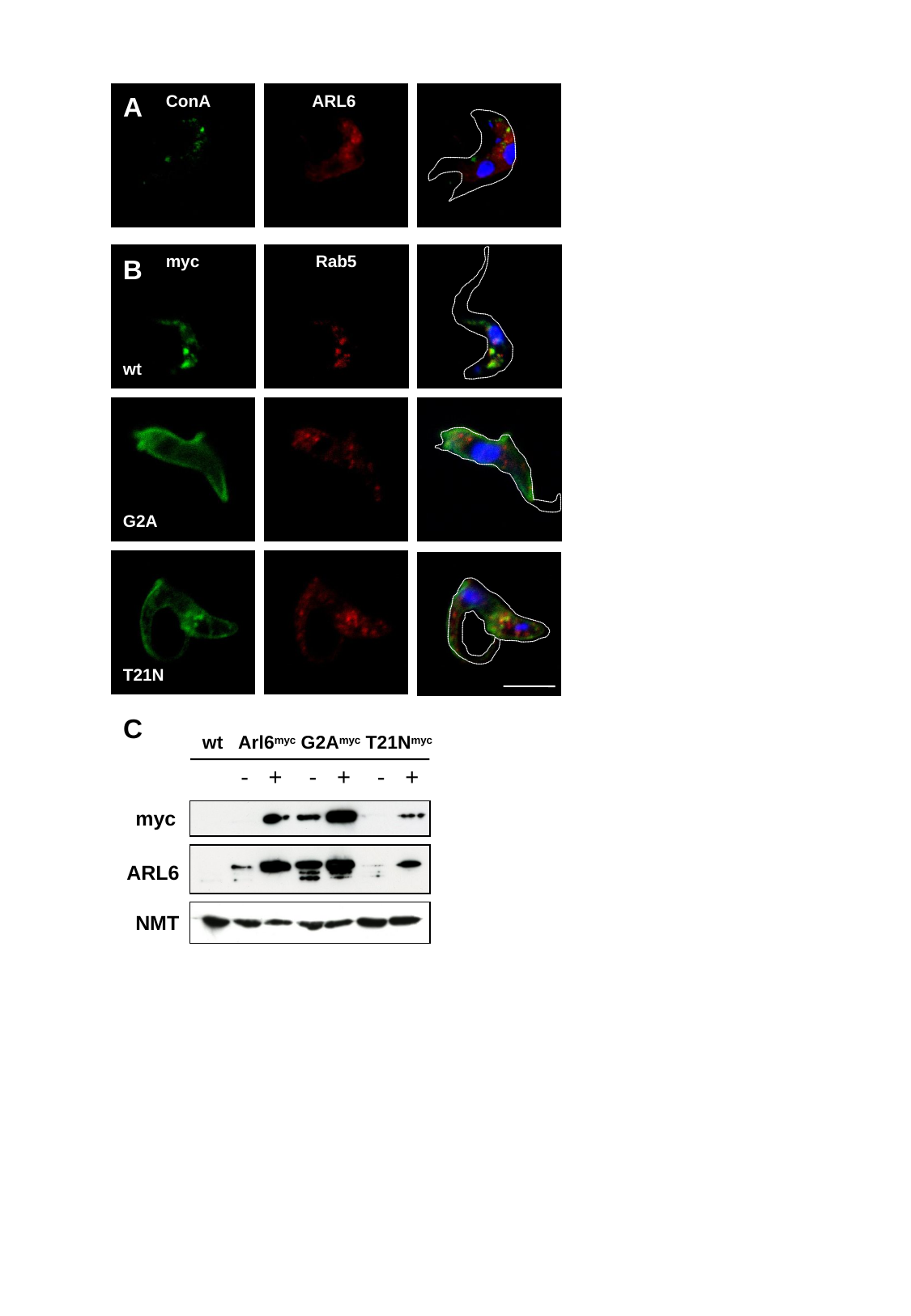

ConA ARL6
A
 myc Rab5 Merge
B
wt
G2A
T21N
C
wt Arl6myc G2Amyc T21Nmyc
- + - + - +
myc
 ARL6
NMT

Supplement: Supplementary Fig. 2 — Localisation of endogenous and mutant forms of T. brucei ARL6. (A) Immunofluorescence analysis of T. brucei bloodstream form (BSF) line Lister 427 pre-treated with Alexa Fluor488 conjugated ConA (green) at 15 °C to stain the flagellar pocket and early endosomes, prior to fixing. Cells were then probed with anti-TbARL6 (red) and co-stained with DAPI (blue). (B) Immunofluorescence analysis of BSF transfected lines 427/pTbARL6MYC, 427/pTbARL6-G2AMYC and 427/pTbARL6-T21NMYC grown in the presence of tetracycline for 24 h. Cells were probed with mouse anti-myc (green) and rabbit anti-TbRab5 (red) and co-stained with DAPI (blue). Bar, 5 μm. (C) Total cell lysates (5 × 10^6^ cells/lane) from BSF parental line Lister 427 (wt) and BSF transfected lines 427/pTbARL6MYC, 427/pTbARL6-G2AMYC and 427/pTbARL6-T21NMYC grown in the absence (−) or presence (+) of tetracycline for 24 h were immunoblotted and probed with mouse anti-myc, anti-TbARL6 and anti-NMT to monitor equal sample loading. [file mmc2.ppt]

## Slide 1
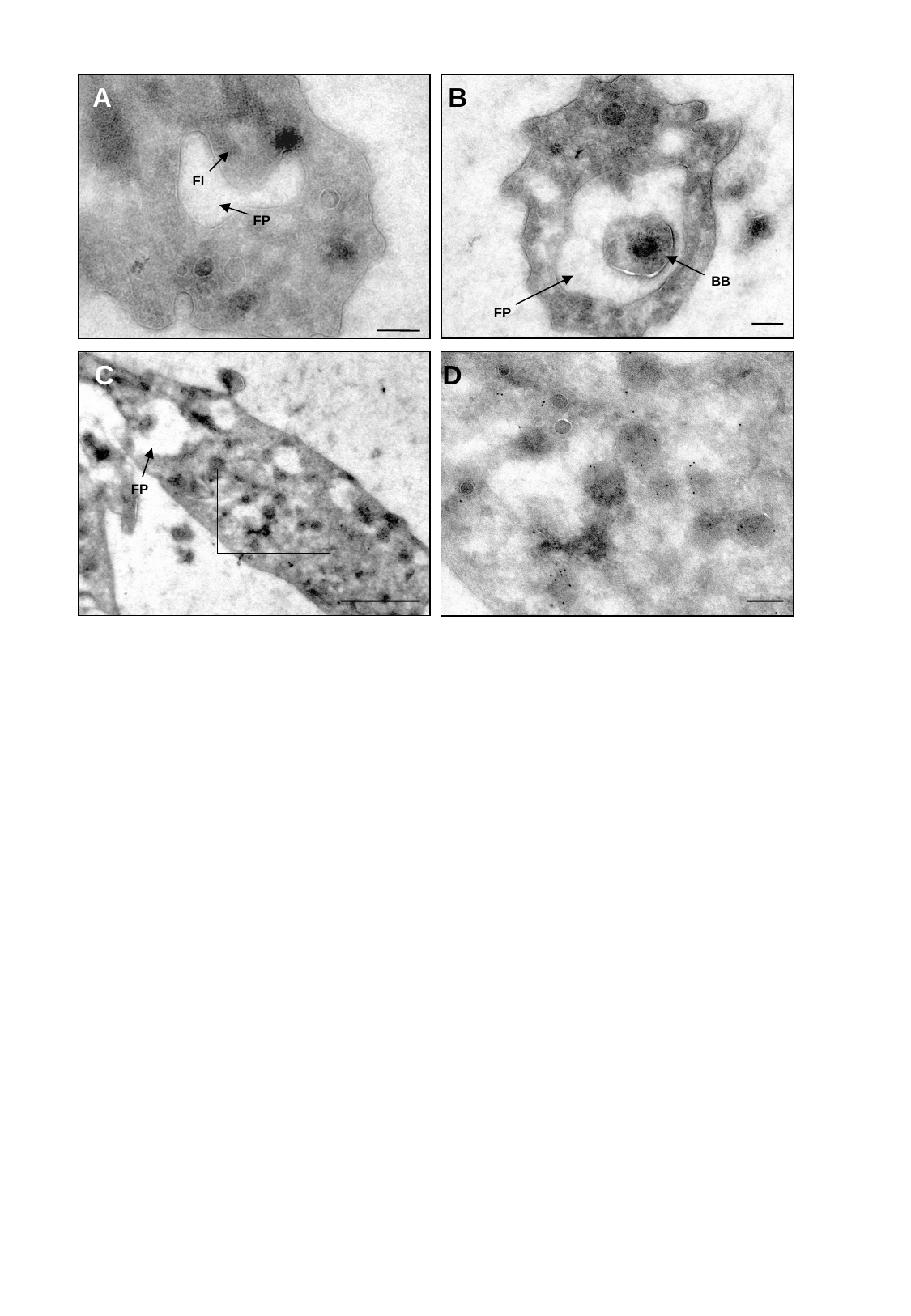

A B
Fl
FP
BB
FP
FP
C D

Supplement: Supplementary Fig. 3 — Transmission immuno-electron micrographs of T. brucei BSF parasites. (A) Parental line Lister 427 probed with 10 nm colloidal gold conjugated goat-anti-rabbit only as a negative control for the data shown in Fig. 1. (B–D) BSF transfected line 427/pTbARL6MYC probed with mouse anti-myc (C–D) or no primary antibody control (B) followed by detection with 10 nm colloidal gold conjugated goat-anti-mouse. Image D is an enlarged view (× 3.1) of the boxed area in image C. BB, basal body. Fl, flagellum. FP, flagellar pocket. Bar, 200 nm (A, B, D) or 1 μm (C). [file mmc3.ppt]

## Slide 1
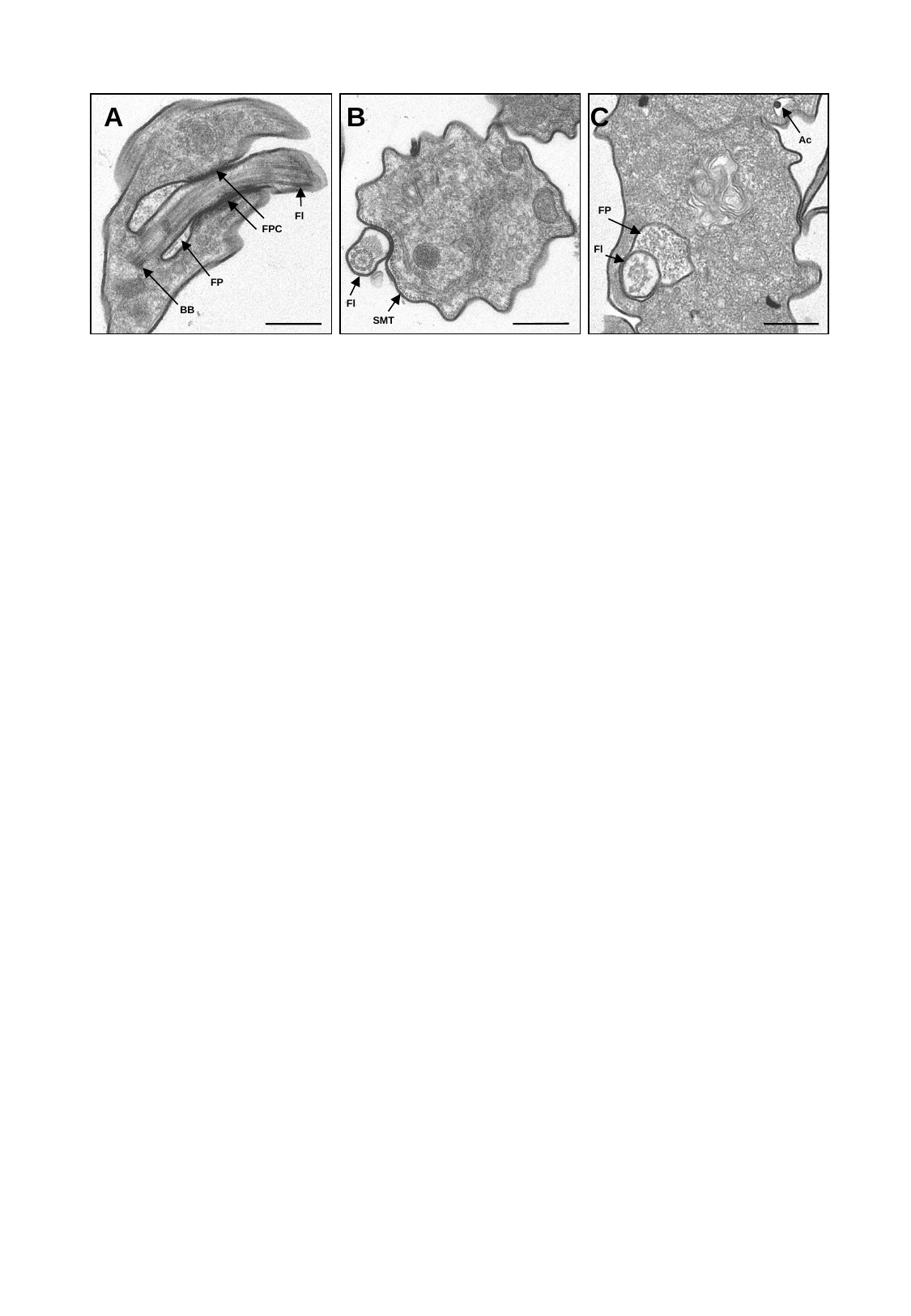

A B C
Fl
FPC
FP
BB
Ac
FP
Fl
Fl
SMT

Supplement: Supplementary Fig. 4 — Transmission electron micrographs of cell line 427/p2T7ARL6 (RNAi) grown in the presence of tetracycline for 24 h. Fl, flagellum. FP, flagellar pocket. FPC, flagellar pocket collar. BB, basal body. SMT, subpellicular microtubules. Ac, acidocalcisome. Bar, 500 nm. [file mmc4.ppt]
