# Supplementary material for: A role for the vesicle-associated tubulin binding protein ARL6 (BBS3) in flagellum extension in Trypanosoma brucei
Source: Biochim Biophys Acta. 2012 Jul;1823(7):1178–91. doi: 10.1016/j.bbamcr.2012.05.007 (PMC3793860; doi:10.1016/j.bbamcr.2012.05.007)
Supplement: Supplementary Fig. 5 — Alignment of kinetoplastid ARL6 orthologues and related protein sequences. Grey shaded areas indicate Switch I and II effector domains. The amino acid residues corresponding to Thr 31 and Gln 71 (human Arf1 numbering) are shown in yellow shading. Sequence accession numbers (GenBank/TriTrypDB): Trypanosoma brucei, Tb927.8.5060; Trypanosoma cruzi, Tc00.1047053508839.60; Leishmania major, LmjF16.1380; Human, AAH24239; Orangutan, NP_001127054.1; Dog, E2RRS0-CANFA; Mouse, AAC62194; Rat, AA162029; Danio rerio, AAI65137; C. elegans, CAA86319; T. brucei ARF1 (TbARF1), Tb09.211.4480; Human Arf1, P84077.2. Human–Long represents the long isoform of Arl6 described in Pretorius et al. (2010). [file mmc5.doc]

Tbrucei ----------MGQSKTKLQVVMCGLDNSGKTTIINQVKPAQSSSKHITATVGYNVETFEK 50

Tcruzi ----------MGQSKTKLHIITCGLDNSGKSSIINRLKPTALQSEHISATVGYNVEVFEK 50

Lmajor ----------MGQAKTKLNIIICGLDNSGKTTIINFMKPENQRSENIAATVGYNVDSFKK 50

Human MGLLDRLSVLLGLKKKEVHVLCLGLDNSGKTTIINKLKPSNAQSQNILPTIGFSIEKFKS 60

Orangutan MGLLDRLSILLGLKKKEVHVLCLGLDNSGKTTIINKLKPSNAQSQNILPTIGFSIEKFKS 60

Human-Long MGLLDRLSVLLGLKKKEVHVLCLGLDNSGKTTIINKLKPSNAQSQNILPTIGFSIEKFKS 60

Dog MGLLDRLSGLLGLKKKEVHVLCLGLDNSGKTTIINKLKPSNAQSQDIVPTIGFSIEKFKS 60

Mouse MGLLDRLSGLLGLKKKEVHVLCLGLDNSGKTTIINKLKPSNAQSQDIVPTIGFSIEKFKS 60

Rat MGLLDRLSGLLGLKKKEVHVLCLGLDNSGKTTIINKLKPSNAQVQDIVPTIGFSIEKFKS 60

Danio MGLFDKLAGWLGLKKKEVNVLCLGLDNSGKTTIINQLKPSNAQAQDIVPTIGFSIEKFKT 60

Celegans MGFFSSLSSLFGLGKKDVNIVVVGLDNSGKTTILNQLKTPETRSQQIVPTVGHVVTNFST 60

TbARF1 MGQWLASAFKSLVGKQEVRILMVGLDAAGKTTILYKLKLGEIVTT--IPTIGFNVETVEY 58

hARF1 MGNIFANLFKGLFGKKEMRILMVGLDAAGKTTILYKLKLGEIVTT--IPTIGFNVETVEY 58

* .:.:: *** :**::*: :* .*:*. : ..

Tbrucei GRVAFTVFDMGGAKKFRGLWETYYDNIDAVIFVVDSSDHLRLCVVKSEIQAMLKHEDIRR 110

Tcruzi GSAKFTVFDMGGAKKFRGLWETYYENINGIIFVVDSSDELRLCVVKEEIELMLQHPDIAR 110

Lmajor GNVYITAFDMSGAQKFRGLWESYYSNIDGVVFVIDSSDALRLCVVKDELEQMLNHADL-- 108

Human SSLSFTVFDMSGQGRYRNLWEHYYKEGQAIIFVIDSSDRLRMVVAKEELDTLLNHPDIKH 120

Orangutan SSLSFTVFDMSGQGRYRNLWEHYYKEGQAIIFVIDSSDRLRMVVAKEELDTLLNHPDIKH 120

Human-Long SSLSFTVFDMSGQGRYRNLWEHYYKEGQAIIFVIDSSDRLRMVVAKEELDTLLNHPDIKH 120

Dog SSLSFTVFDMSGQGRYRNLWEHYYKEGQAIIFVIDSSDRLRMVVAKEELDTLLNHPDIKH 120

Mouse SSLSFTVFDMSGQGRYRNLWEHYYKDGQAIIFVIDSSDKLRMVVAKEELDTLLNHPDIKH 120

Rat SSLSFTVFDMSGQGRYRNLWEHYYKDGQAIIFVVDSSDKLRMVVAKEELDTLLNHPDIKH 120

Danio SSLSFTVFDMSGQGRYRNLWEHYYKEGQAIIFVIDSGDKLRMVVAKEELDTLLNHPDIKH 120

Celegans QNLSFHAFDMAGQMKYRSTWESYFHSSQGVIFVLDSSDRLRMELLKDELMMVMEHKDVVS 120

TbARF1 KNLKFTMWDVGGQDVLRPLWRHYYQNTNGIIFVVDSNDKERVGKARQELEKMLSEDELRN 118

hARF1 KNISFTVWDVGGQDKIRPLWRHYFQNTQGLIFVVDSNDRERVNEAREELMRMLAEDELRD 118

: :*:.* * *. *: . :.::**:**.* *: :.*: :: . ::

Tbrucei ELP--GGGRVPFLFFANKMDAAGAKTAAELVEILDLTTLMGDHPFVIFASNALKGTGVHE 168

Tcruzi ELPKTNGAKIPFLFYANKMDLPNAKTAAELVDLLDLTTLMADRPFNIFASNALKGTGVNE 170

Lmajor -----KACTVPFVFFANKMDLAGAKTPMELTQILQLNSLMGDHPMNIFTSNALRGEGIHE 163

Human R-------RIPILFFANKMDLRDAVTSVKVSQLLCLENIK-DKPWHICASDAIKGEGLQE 172

Orangutan R-------RIPILFFANKMDLRDAVTSVKVSQLLCLENIK-DKPWHICASDAIKGEGLQE 172

Human-Long R-------RIPILFFANKMDLRDAVTSVKVSQLLCLENIK-DKPWHICASDAIKGEGLQE 172

Dog R-------RIPILFFANKMDLRDAVTSVKVSQLLCLENIK-DKPWHICASDAIKGEGLQE 172

Mouse R-------RIPILFFANKMDLRDSVTSVKVSQLLCLESIK-DKPWHICASDAIKGEGLQE 172

Rat R-------RIPILFFANKMDLRDAVTSVKVSQLLCLENIK-DKPWHICASDALKGEGLQE 172

Danio R-------RIPLLFFANKMDLRDALSAVKVSQLLCLENIK-DKPWHICASDAVKGEGLLE 172

Celegans R-------GIPIVILANKMDIPGAMTASDITVALGLNLYR-SGTWSIHSTCALTGDGLDK 172

TbARF1 A---------VLLVFANKQDLPNAMSTTEVTEKLGLQSVR-QRNWYIQGCCATTAQGLYE 168

hARF1 A---------VLLVFANKQDLPNAMNAAEITDKLGLHSLR-HRNWYIQATCATSGDGLYE 168

::. *** * .: .. .: * * * * . *: :

Tbrucei GFSWLQETASRQSG--KAGTKRG----- 189

Tcruzi GFFWLQNALLRQSNTISSSSSRGARQRN 198

Lmajor GMDWLRSMMLRRLE---QSKKK------ 182

Human GVDWLQDQ-------IQTVKT------- 186

Orangutan GVDWLQDQ-------IQTVKT------- 186

Human-Long GVDWLQEKQYKSDPDCEDMKR------- 193

Dog GVDWLQDQ-------IQAVKT------- 186

Mouse GVDWLQDQ-------IQAVKT------- 186

Rat GVDWLQDQ-------IQAVKT------- 186

Danio GVDWLQDQ-------IRAMKT------- 186

Celegans AMQQLSAEITK----YMESRRT------ 190

TbARF1 GLDWLSAN-------IKKSMK------- 182

hARF1 GLDWLSNQ-------LRNQK-------- 181

.. *
