# Supplementary material for: A role for the vesicle-associated tubulin binding protein ARL6 (BBS3) in flagellum extension in Trypanosoma brucei
Source: Biochim Biophys Acta. 2012 Jul;1823(7):1178–91. doi: 10.1016/j.bbamcr.2012.05.007 (PMC3793860; doi:10.1016/j.bbamcr.2012.05.007)
Supplement: Supplementary Table 1 — List of primer sequences. [file mmc6.doc]

| **Primer** | **Sequence** |
| --- | --- |
| ARL6-F1 | 5’-CACCACCACCACATGGGACAATCCAAAACGAAGCTGCAAG-3’ |
| ARL6-R1 | 5’-GAGGAGAAGGCGCGTCAGCCCCTCTTCGTCCCAGC-3’ |
| ARL6-F2 | 5’-AAGCAGAAGAAGCTTACCGCG-3’ |
| ARL6-R2 | 5’-GCTGTGTCTAGAGCCCCTCTTC-3’ |
| ARL6-F3 | 5’-AGCTTACCGCGGCGATGGCACAATCCAAA-3’ |
| ARL6-R3 | 5’-CATCGCCGCGGTAAGCTTTGAATTTTAC-3’ |
| ARL6-F4 | 5’-GCTAGACAACAGTGGGAAAAATACAATTATTAA-3’ |
| ARL6-R4 | 5’-TTTTCCCACTGTTGTCTAGCCCGCACATGA-3’ |
| ARL6-F5 | 5’-CATGTGCGGTCTAGATAACAGTGGGAAAAC-3’ |
| ARL6-R5 | 5’-GCAAAATCTAGAAACGGCACCCGA-3’ |
| ARL6-F6 | 5’-AAAAGGGCCCATGGGACAATCCAAAACGAAG-3’ |
| ARL6-R6 | 5’-AAAAGCGGCCGCCCCCTCTTCGTC-3’ |
| BBS1-F1 | 5’-GATTGTTATTATTAGTCTAGAATGCCTTTCTTTTCCTTG-3’ |
| BBS1-R1 | 5’-CGATCAGGGATCCACCTCTATGAATTGTCTTC-3’ |
